# DFT and molecular simulation validation of the binding activity of PDEδ inhibitors for repression of oncogenic k-Ras

**DOI:** 10.1371/journal.pone.0300035

**Published:** 2024-03-08

**Authors:** Taghreed A. Majrashi, Ahmed Sabt, Hadia Almahli, Mahmoud A. El Hassab, Mahmoud A. Noamaan, Eslam B. Elkaeed, Mohamed Farouk Hamissa, Abdalkareem Nael Maslamani, Moataz A. Shaldam, Wagdy M. Eldehna

**Affiliations:** 1 Department of Pharmacognosy, College of Pharmacy, King Khalid University, Asir, Saudi Arabia; 2 Chemistry of Natural Compounds Department, Pharmaceutical and Drug Industries Research Institute, National Research Centre, Dokki, Cairo, Egypt; 3 Department of Chemistry, University of Cambridge, Cambridge, United Kingdom; 4 Faculty of Pharmacy, Department of Medicinal Chemistry, King Salman International University (KSIU), South Sinai, Egypt; 5 Faculty of Science, Mathematics Department, Cairo University, Giza, Egypt; 6 Department of Pharmaceutical Sciences, College of Pharmacy, AlMaarefa University, Ad Diriyah, Riyadh, Saudi Arabia; 7 Medicinal and Pharmaceutical Chemistry Department, Pharmaceutical and Drug Industries Research Division, National Research Centre, Dokki, Giza, Egypt; 8 Institute of Organic Chemistry and Biochemistry, Academy of Sciences, Prague, Czech Republic; 9 Faculty of Medicine, Cairo University, Cairo, Egypt; 10 Faculty of Pharmacy, Department of Pharmaceutical Chemistry, Kafrelsheikh University, Kafrelsheikh, Egypt; Universiti Teknologi Malaysia, MALAYSIA

## Abstract

The development of effective drugs targeting the K-Ras oncogene product is a significant focus in anticancer drug development. Despite the lack of successful Ras signaling inhibitors, recent research has identified PDEδ, a KRAS transporter, as a potential target for inhibiting the oncogenic KRAS signaling pathway. This study aims to investigate the interactions between eight K-Ras inhibitors (deltarazine, deltaflexin 1 and 2, and its analogues) and PDEδ to understand their binding modes. The research will utilize computational techniques such as density functional theory (DFT) and molecular electrostatic surface potential (MESP), molecular docking, binding site analyses, molecular dynamic (MD) simulations, electronic structure computations, and predictions of the binding free energy. Molecular dynamic simulations (MD) will be used to predict the binding conformations and pharmacophoric features in the active site of PDEδ for the examined structures. The binding free energies determined using the MMPB(GB)SA method will be compared with the observed potency values of the tested compounds. This computational approach aims to enhance understanding of the PDEδ selective mechanism, which could contribute to the development of novel selective inhibitors for K-Ras signaling.

## 1. Introduction

Ras proteins, also known as small GTPases, play a vital role in the signaling network that regulates the differentiation of cells, proliferation, or survival by participating in Ras-Raf-MAPK pathway [1,2]. Oncogenic alterations in particular amino acids, including amino acids corresponding to the codons 12, 13, and 61, are responsible for maintaining Ras in the GTP-bound form [3,4]. This results in anomalous signaling, which leads to illnesses such as tumor [5,6]. Despite extensive attempts, no direct small-molecule signaling inhibitor medication for mutant RAS has been marketed [7]. There is a high probability of K-Ras mutation in pancreatic tumors and 45% in colorectal tumors, as well as 30% in lung tumors [4,8]. Therefore, choosing the appropriate and effective therapeutic strategy against K-Ras mutant is considered of fundamental importance in medicinal chemistry [9,10].

The progression of signal transduction is contingent upon the optimal concentration of k-Ras protein centered on the membrane of the plasma (PM) [11–13]. Normally, this step is antagonized by entropic equilibration to the extensive surface of the endomembrane [10,14]. However, proteins that promote the solubilization of guanine nucleotide dissociation inhibitors (GDI), such as shuttling protein PDEδ, prevent this equilibration through binding to farnesyl moiety of k-Ras and minimizing its attachment to the internal membrane and thereby making it available to diffuse throughout the cell [15–18]. At the plasma membrane, GTP-bound Arf-like protein 2 (Arl2) is the release factor that contributes to the release of k-Ras from the PDEδ in perinuclear membranes, which is held by electrostatic interaction on the recycling endosome and then returned through vesicular transport to the PM [19]. Therefore, any disturbance in this cycle, by blocking the attachment site of k-Ras in PDEδ, could offer a different treatment approach for oncogenic signaling of KRAS [20].

**Fig 1:**
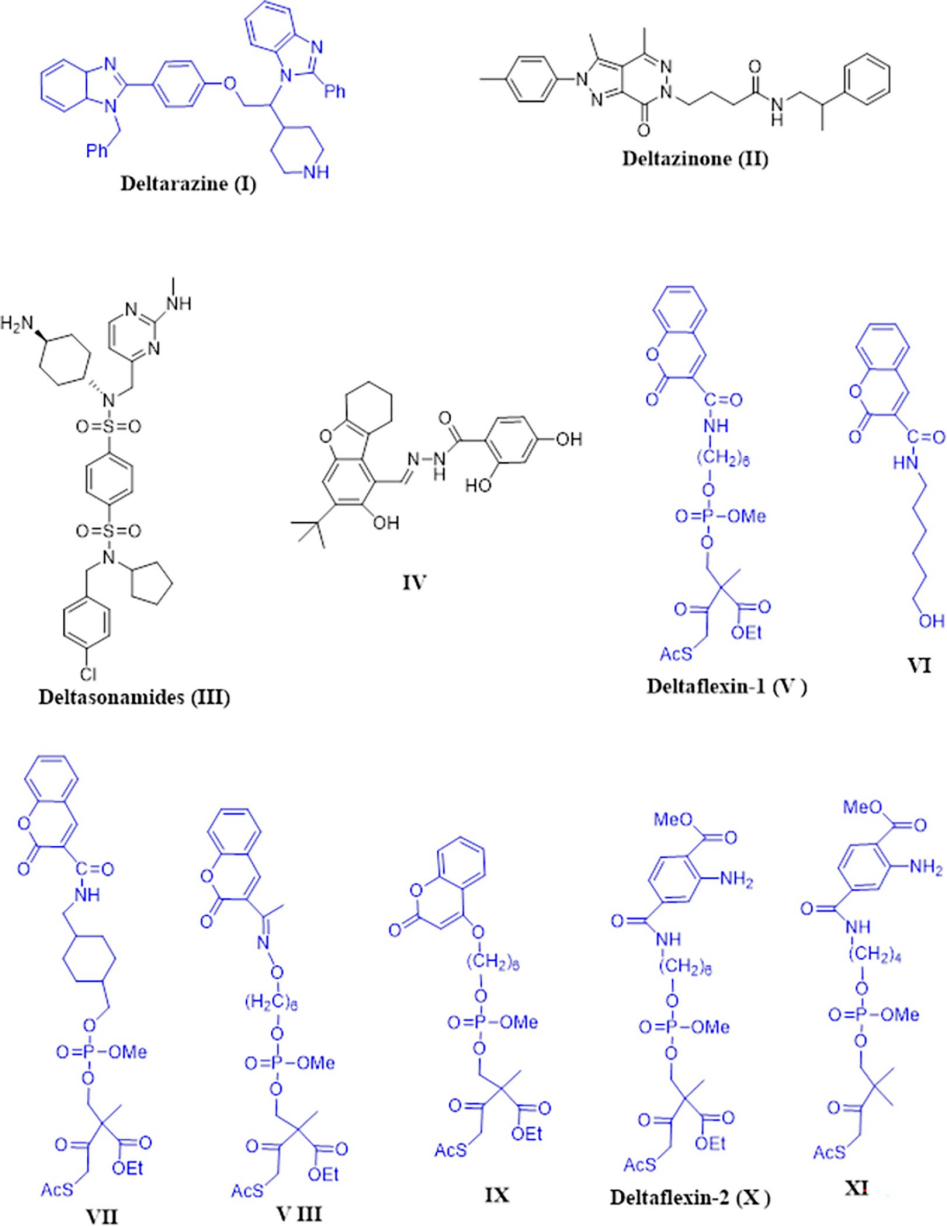
Structure for some reported KRAS‒PDEδ inhibitors.

**Deltarasin (I**, *K*_D_ 38 ± 16 nM**)** and **Deltazinone (II,** *K*_D_ 8 ± 4 nM**)** were the first and second generations developed by the Waldmann group to be PDEδ inhibitors with a high level of affinity, both *in vitro* and *in vivo*, at the nanomolar scale **(Fig 1)**. Unfortunately, the major problem that appeared by both inhibitors is the release by Arl2 besides the cytotoxicity by **Deltarasin** at high concentrations (5μM) [21,22]. **Deltasonamides (III),** representing the third generation of PDEδ inhibitors, could significantly withstand Arl2-mediated ejection because these molecules bind to PDEδ and form up to seven hydrogen bonds with a high affinity in the picomolar range, leading to decreased release by Arl2 [15]. Despite this, these compounds are poorly partitioned and penetrate poorly into cells. Other compounds were reported to block PDEδ and hence suppress the oncogenic k-Ras, such as premonensin-derivatives [23] besides compound **IV** which was uncovered by Leung *et al* in 2019 [24]. Through breaking the K-Ras/PDE attachment, as a result of NHTD treatment, less proliferation and apoptosis occurred in NSCLC having mutated K-Ras [25]. In 2020, Abankwa and coworkers developed a new model for inhibiting PDEδ called Deltaflexin 1, 2 and their derivatives **(V-XI)** which is characterized by selectivity against k-Ras not H-Ras. They possessed potent anti-proliferative activity on breast and colorectal tumors, as well as the ability to suppress lung and breast cancer stemness traits [15]. These compounds were able to repress around a 1000 times difference in effectiveness between *in vitro* and in cellulo due to presence of biodegradable cell penetration group [26,27]. It was proposed that key interactions between the hydrophobic cavity of PDEδ and the farnesyl group of Ras family alongside interactions with Arg61 and Tyr149 can put the inserted molecule into the receptor’s binding site of PDEδ’s in the correct orientation [28,29]. Hydrophobic interactions with Met117 and Glu88 and hydrogen bond interaction with Glu78 are considered useful regarding the selectivity and affinity of PDEδ inhibitors [15,23,24,30].

Consequently, the aforementioned data may be utilized to develop rationally designed new PDEδ inhibitors and encourage us to study these compounds *via* combined computational approaches. This approach has been reported to design different inhibitors [31–34]. The molecular structural features of target derivatives, their importance in relation to drug-like characteristics, and the way they interact were investigated using advanced computational methods such as DFT/wB97XD, high-level calculations were performed. The wave function representation was expanded using 6–311++G(d,p) as the basis sets. The method of DFT was employed by using wB97XD functional with 6–311++G(d,p), which can replicate empirical geometrical outcomes for computational investigations on interested eight active form derivatives. Chemical descriptors, such as chemical hardness (h), chemical potential (m), and electrophilicity (w), were computed using the functionality of wB97XD in conjunction with the 6–311++G(d,p) basis set. These descriptors were employed to discern alterations in reactivity. Using a multidimensional charge-based approach, researchers were able to understand how these compounds interact with the biologically active of the oncogenic K-Ras receptors. The study focused on analyzing the molecular electrostatic surface potential (MESP) to understand how charges are distributed on a molecule. The goal was to identify areas with potential for hydrogen bonding, as well as regions with electrophilic and nucleophilic properties. Through the utilization of reliable and accurate data and effective visual representation, we successfully identified the site of activity in these derivatives and predicted binding site and binding energy through chemical docking analysis. Investigation of the correlation between the molecular structure and the biological activity of the compounds suggested that they have favorable properties for oral bioavailability. Furthermore, we conducted MD simulations for 100 ns to examine both stability and dynamics of the ligand-receptor complexes.

## 2. Results and discussion

### 2.1. Density functional theory (DFT)

#### 2.1.1. Molecule orbital calculations

Natural charges, energetics of the ground state, molecular electrostatic potential maps, naturally occurring populations of the nucleus of the suggested derivatives, also the molecular properties and reactivity descriptors of the investigated compounds were computed and examined.

**Fig 2:**
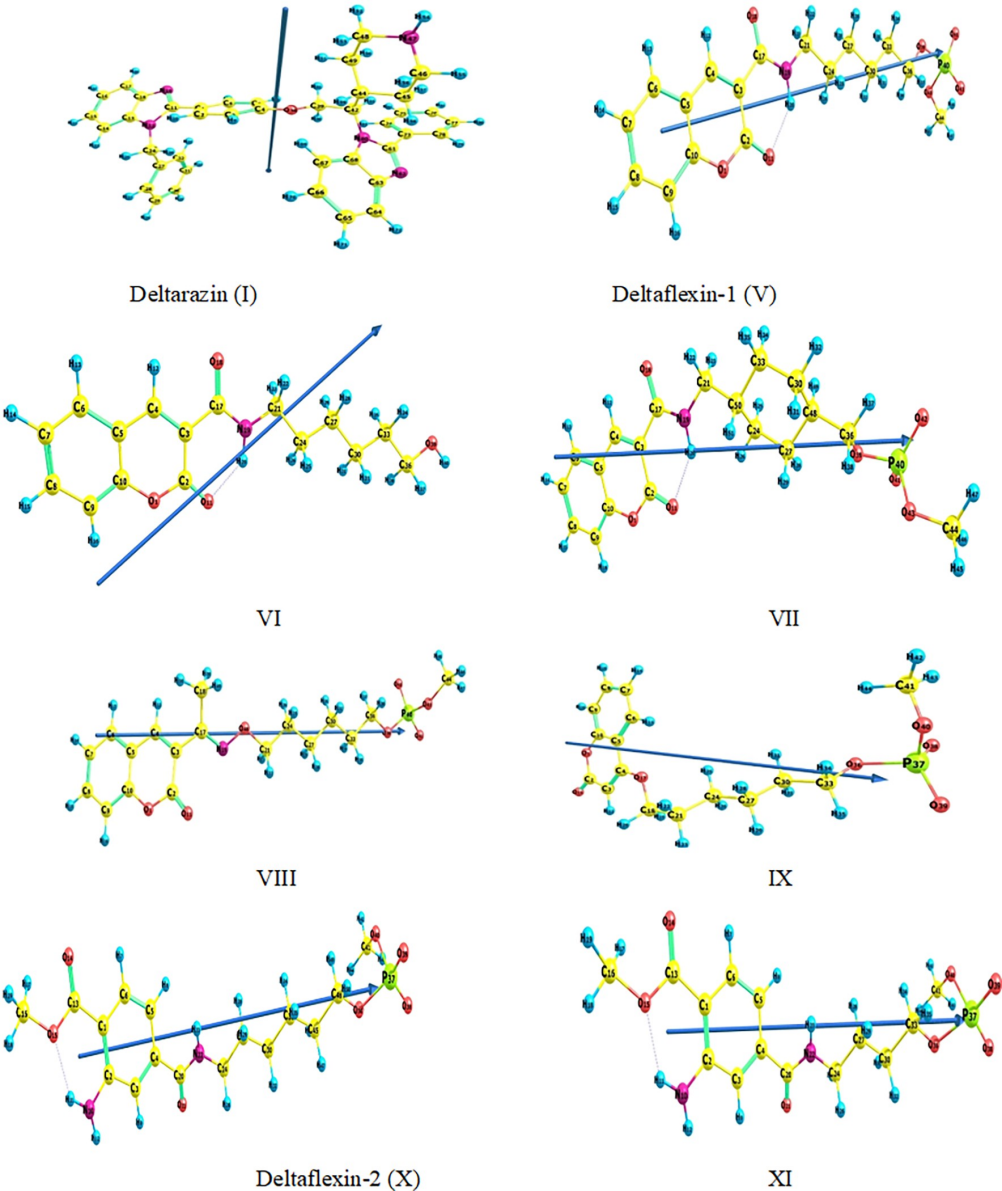
The optimal shape and momenta of the dipole vector of the potential eight target molecules (I, V-XI) were determined utilizing the wB97XD useful and 6–311++g(d,p) basis set.

*2*.*1*.*1*.*1*. *Methods benchmark*. The selection of computational approach and basis set significantly impacts the precision of calculations. The optimization of the molecular geometry of a specific hybrid coumarin derivative (VI) **(refer to Fig 2)** was conducted using three distinct levels of theoretical methods. From the beginning/without electron correlation, the wavefunction Schrodinger equation is used in non-electron correlated HF, along with hybrid DFT (B3LYP) [2–5] and long-range corrected (LC) DFT (wB97XD) [6], which uses the electron density approach. These methods utilize two distinct basis sets: 6–311++G (d, p) and 6–311+G (d) [7]. The table in Supplementary data, S1 Table, presents the bond lengths obtained from geometry optimization. These results are then compared with the bond lengths derived from x-ray diffraction of 3-((2-Oxo-2H-chromen-3-yl)carbonyl) pyridinium hydrogen squarate (ref. CCDC 697425)[8] in order to identify the most effective computational method for reproducing the experimental x-ray geometrical parameters. **Table 1** displays a comparison of bond lengths for a specific hybrid coumarin derivative compound (VI) calculated using different methods and the 6–311++G (d, p) and 6–311+G (d) basis sets. The findings indicate that the LC-DFT (wB97XD) method demonstrates closer alignment with experimental data [8] compared to the ab initio HF and hybrid B3LYP methods. This is evident from the mean absolute deviation A% values, which are computed as the average absolute differences between the calculated and experimental values for each method and basis set, as presented in the final row of **Table 1**. The findings suggest that the LC-DFT/wB97XD/6-311++G (d, p) method is the most appropriate for estimating the bond length of the chosen hybrid coumarin derivative (VI). As a result, it is utilized for the remaining calculations in this study.

**Table 1:** Displays the experimental bond length and calculated values for a specific hybrid coumarin derivative (VI) at various calculation levels.

Parameters	Exp. [8]	6–311++G(d,p)			6–311+G(d)		
		*ab initio*/*HF*	*DFT*/ *B3LYP*	*LC-DFT*/wB97XD	*ab initio*/*HF*	*DFT*/ *B3LYP*	*LC-DFT*/wB97XD
R(O1,C2)	1.438	1.346	1.377	1.370	1.346	1.378	1.370
R(O1,C10)	1.458	1.349	1.369	1.357	1.348	1.362	1.357
R(C2,C3)	1.324	1.472	1.466	1.466	1.472	1.466	1.470
R(C2,O11)	1.328	1.185	1.209	1.206	1.185	1.209	1.206
R(C3,C4)	1.379	1.339	1.348	1.350	1.339	1.349	1.350
R(C3,C17)	1.435	1.512	1.515	1.513	1.511	1.515	1.513
R(C4,C5)	1.397	1.443	1.439	1.433	1.443	1.438	1.433
R(C5,C6)	1.425	1.398	1.403	1.403	1.398	1.403	1.403
R(C5,C10)	1.330	1.384	1.404	1.396	1.384	1.404	1.396
R(C6,C7)	1.313	1.375	1.384	1.380	1.375	1.384	1.380
R(C7,C8)	1.367	1.396	1.403	1.400	1.396	1.403	1.400
R(C8,C9)	1.401	1.378	1.381	1.383	1.379	1.381	1.380
R(C9,C10)	1.294	1.386	1.398	1.391	1.386	1.398	1.391
R(C17,O18)	1.254	1.214	1.224	1.222	1.213	1.224	1.222
A%		**5.125**	**4.819**	**4.755**	**5.127**	**4.847**	**4.791**

$A\%=\frac{Mean\;absolute\;deviation\;of\;bond\;length}{Mean\;bond\;length\;of\;experemential\;values\;}x100$.

#### 2.1.2 Ground state geometric parameters

This study aimed to provide a comprehensive analysis of the optimized geometry, dipole moment vector, numbering system, bond angles, bond lengths, and dihedral angles for all selected hybrid coumarin derivatives **(V-IX)**, **S1 Table** (**Supplementary data**). These parameters will serve as a benchmark for evaluating the findings of this research. We have chosen specific geometric parameters to contrast the gas-phase wB97XD /6-311++G(d,p) calculations with the crystal data obtained from the X-ray structure of 3-((2-Oxo-2H-chromen-3-yl)carbonyl) pyridinium hydrogen squarate (ref. CCDC 697425) [35]. The computed mean absolute errors (MAEs) for the coumarin scaffold’s selected lengths of bonds and angles are presented in **S2 Table (see supplementary data).** The MAEs range for bond length was between 0.017 and 0.356 Å, and for bond angles were between 0.0 and 2.32 degrees, and for dihedral angles were between 0.09 and 2.16 degree when using wB97XD functional. In the **V-IX** derivatives, most of the measured lengths of bonds indicate underestimations between 0.25 and 1.1% in O1-O11 and overestimations between 1.1 and a 5.1% increase in C8-O18. Overall, there is a significant difference observed in the data from experimental finding in bond length. Analyzing the angle of the dihedral observations provided in **S2 Table (see supplementary data)** shows that almost all molecules are planar except for N19-alkyl, an (PO_4_)^-2^ moieties. All the selected compounds **(V-IX)** have out-of-plane components, with diffraction angles ranging from -39.3 to -42.5 degrees. The bond angles have that been determined through calculations (**S2 Table**) vary between 109.0 to 125.0 degrees, which compare nicely to a regular SP^3^ and SP^2^ hybridization geometry, respectively.

In addition, **S3 Table (see supplementary data)** presents the geometry optimization, numbering system, dipole moment vector, length of bonds, angles of bonds, and dihedral angles for **Deltarazin (I)**, **Deltaflexin-2 (X)**, and **XI** compounds (**Fig 2)**, where the computations were carried out using the wB97XD/6-311++G(d,p) technique as the most accurate measure of theory. **Deltarazin (I)** contained C, N aromatic, and non-aromatic rings with single-double resonance, with bond length varying from 1.3 to 1.5 Å and bond angle varying from 105.11 to 133.0 degrees which is related to the basic concepts of hybridization of molecular orbitals. In dihedral angles, the C1-left arm of **Deltarazin** D(C6, C1, C11, N12) out of plane by 45 degrees. Benzyl moiety also out of plane D(C1,C11,N12,C24), D(C11,N12,C24,C27), D(N12,C24,C27,C32), D(O38,C39,C42,N60), D(N60,C42,C44,C45), D(C44,C45,C46,N47) and D(N60,C61,C73,C74) are 134.14, 15.05, 100.16, 80.0, 54.36, 56.55 and 47.6 degrees, respectively. **Deltaflexin-2** (**X**) and **XI** compounds are so close together in geometrical structure planarity of the phenyl group with consistency in bond length, angle, and dihedral angle of C4-amide alkyl group ended by PO_4_ that is out of plane, and this compare nicely to a regular SP^2^ and SP^3^ hybridization geometry, respectively.

#### 2.1.3 Natural charges and natural population

The target compounds’ **(I, V-XI)** natural charge analysis clearly demonstrates the arrangement of electrons throughout the different subshells that constitute their orbitals at the atomic level. **S4 Table (see supplementary data)** displays the charge assessment for all target compounds utilizing 6–311++G(d,p) basis set and the wB97XD functional. The electronegative charges for (**V-XI**) are accumulated on O42, O41, O43, and O39 of the phosphate anion center, O1, O11 of the lactam ring of the coumarin scaffold, O18 and N19 of the amide group attached to C3, respectively, having values between -1.163 e and -0.591 e, and tending to donate the electrons. In the case of compound (**I**), charges are accumulated on N47, O38, N19, N62, N60 and N12, respectively, with values from -0.685 e to -0.46 e.

However, the electropositive atoms for (**V-XI**), such as P40, C2, C17, H20, C10 and H12, respectively have values between +2.458 e and +0.238 e and tend to accept electrons. Moving from **I** to **XI** involves a slight alteration in the inherent charge while maintaining the same sequential pattern in the electrostatic projection sequence. A thoroug examination of the inherent charge distribution of the active eight target compounds is extremely beneficial in understanding the interaction between these ligands and their targets.

#### 2.1.4. Frontier molecular orbitals (FMOs) analysis

According to the information provided in **Table 2**, **Deltarazin (I)** exhibited greater stability and lower reactivity,h in comparison with other possible hybrids, with an energy gap value of 8.39 eV. In contrast, the hybrid **Deltaflexin-1 (V)**, with an energy gap of 4.77 eV, exhibited the least stability and the greatest reactivity [36–38]. The remaining active forms had their energy gaps arranged in the following order: **VII**<**VIII**<**Deltaflexin-2** (**X**)<**IX**<**XI**<**VI**.

**Table 2:** Energetic parameters of selected potential eight target compounds (I, V-XI) by utilizing wb97xd/6-311++g(d,p).

Parameters	ET^1^	EHOMO^1^	ELUMO^1^	Eg^2^	Μ^3^	I^2^	A^2^
Deltarazin (I)	-1895.40573	-0.28985	0.01838	8.39	0.84	7.89	-0.50
Deltaflexin-1 (V)	-1583.26976	-0.16344	0.01172	4.77	40.64	4.45	-0.32
VI	-976.77624	-0.32911	-0.02938	8.16	3.80	8.96	0.80
VII	-1660.69842	-0.16625	0.01813	5.02	33.56	4.52	-0.49
VIII	-1622.48461	-0.16396	0.02195	5.06	42.07	4.46	-0.60
IX	-1489.79107	-0.17855	0.04865	6.18	26.51	4.86	-1.32
Deltaflexin-2 (X)	-1601.74485	-0.17599	0.04218	5.94	40.30	4.79	-1.15
XI	-1523.12089	-0.17868	0.05102	6.25	33.64	4.86	-1.39

^1^ Data in (au) unit.

^2^ Data in (eV) unit.

^3^ Data in (D) unit.

**Fig 3:**
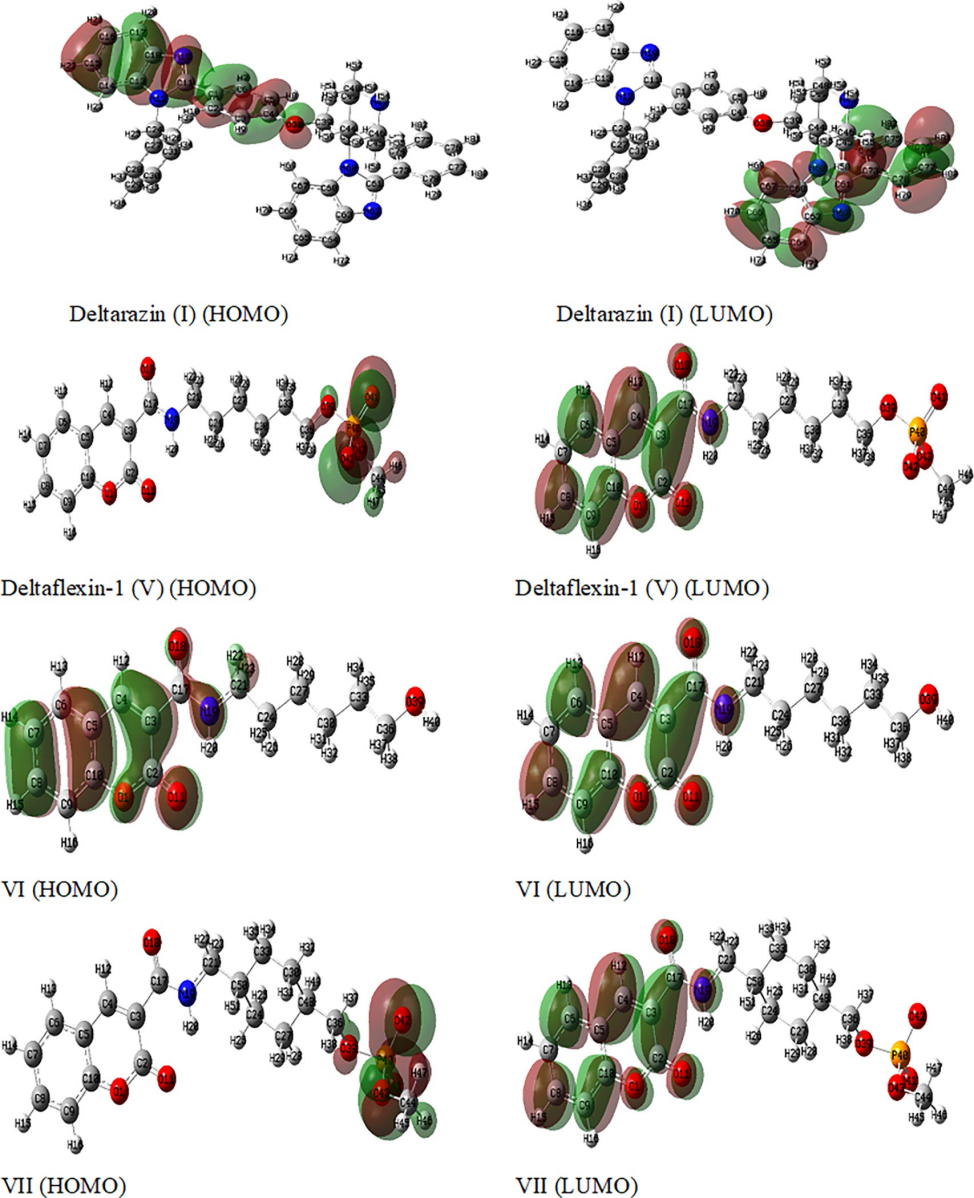
The frontier orbitals of molecules of the eight probable target compounds (I, V-XI) were examined utilizing the computational approach wb97xd/6-311++g(d,p).

Calculating the potential for ionization (I) and affinity for electrons (A) parameters enabled the estimation of the global reactivity descriptors, and this corresponds to the values of energy in (HOMO) and (LUMO) [39,40]. As stated by the data **(Table 2),** compound **VI** exhibited the greatest values of I (8.96 eV) and A (0.8 eV). The sequence of the **I** values for the compounds is as follows. **I**>**XI**>**IX**>**X**>**VII**>**VIII**>**V**. Conversely, the sequence for A values is as follows: **V**>**VII**>**I**>**VIII**>**X**>**IX**>**XI**. Derivatives **I, V, and X** exhibit strong potential as suitable candidates for engaging in interactions with other biological mechanisms that repress the activity of oncogenic k-Ras receptors. All the potential inhibitors exhibit nearly identical dispersion of HOMO and LUMO isodensity, as illustrated in **Fig 3**, with the exception of compounds **I** and **VI**. These two compounds display slight variations in the dispersion patterns on the coumarin cores and the benzimidazole scaffold, respectively. The dipole moment vector, in conjunction with the order norm vector, serves to indicate the pattern of the electronic transference of charge. Consequently, the synthesized compounds can be arranged in a hierarchical order as follows: **I < VI < IX < VII < XI < X < V < VIII.**

#### 2.1.5 Global reactivity descriptors

The density functional theory (DFT) employs the electron density of a chemical system to elucidate fundamental concepts pertaining to chemical reactivity [41]. The global descriptors generated at the wB97XD functional with 6–311++G (d, p) basis set are employed to address a variety of qualitative notions in chemical reactivity [42,43]. Herein, we investigated the reactivity of eight derivatives (**I, V-XI**). **Table 3** shows the values of descriptors which identify their reactivity or stability. Among them, compound **I,** “**Deltarazin,**” displayed the greatest value (η = 4.19 eV), which has chemical stability, while compound **V** showed the minimal value (η = 2.38 eV), indicating it has chemical reactivity. The order of chemical stability for other compounds is **VII>VIII>X>IX>XI>VI**. Electronic chemical potential (V) values reflect the transfer of charges in a molecule’s base state. According to **Table 3**, compound **XI** exhibits the highest value of -1.74 eV, while compound **VI** demonstrates the smallest value of -4.88 eV. The remaining target compounds are ranked as follows: **I, V, VII, VIII, X, and IX**.

**Table 3:** Reactivity indices of selected potential eight target compounds (I, V-XI) using wB97XD functional with 6–311++G (d, p) basis set.

Parameters^1^	X	η	S	V	Ω	N
Deltarazin (I)	3.69	4.19	0.12	-3.69	1.63	-3.68
Deltaflexin-1 (V)	2.06	2.38	0.21	-2.06	0.89	-0.24
VI	4.88	4.08	0.12	-4.88	2.92	-4.74
VII	2.02	2.51	0.20	-2.02	0.81	-0.31
VIII	1.93	2.53	0.20	-1.93	0.74	-0.25
IX	1.77	3.09	0.16	-1.77	0.51	-0.65
Deltaflexin-2 (X)	1.82	2.97	0.17	-1.82	0.56	-0.58
XI	1.74	3.13	0.16	-1.74	0.48	-0.65

^1^ Data in (eV) unit.

The ω index, which measures electrophilicity, is a thermodynamic variable that quantifies the energy variations in a chemical system as it becomes fully saturated with additional electrons. This index serves as a determinant of the reactivity of the chemical system. Compound **XI** exhibits a nucleophilic character, as evidenced by its electrophilicity index value of 0.48 eV, which is the lowest among the compounds studied. On the other hand, compound **VI** is electrophilic in nature (ω = 2.92 eV) **(Table 3)**. The concept of electronegativity (X) is employed to characterize the propensity of an atom within a covalent bond to attract electrons. Compound **VI** exhibits a significantly greater affinity towards X = 4.88 eV. In the electrophilicity indices, the electronegativity (X) order among the other compounds exhibits a similar tendency. In relation to the global level of gentleness (S), compound **V** showed the highest reactivity (0.21 eV), while the remaining samples demonstrated similar levels of softness, ranging from 0.12 to 0.20 eV, in the following order: **VII, VIII, X, IX, XI, VI, and I**.

#### 2.1.6 Local reactivity descriptors

Investigating a molecule’s favored location and chemical reactivity has frequently involved the use of local reactivity characteristics [44,45]. The Fukui function is a tool that can be employed to examine the selectivity of molecular sites at a local level [46]. The equation represents the relationship between the first derivative of the electronic density *ρ*(r) with respect to the number of electrons (N) in a system while keeping the external potential ν(r) constant [47].


$$(r)={\left(\frac{\partial \rho (r)}{\partial N}\right)}_{v(r)}={\frac{1}{2}(\frac{\partial \mu }{\partial v(r)})}_{v(r)},$$

By analyzing the variations in electrical density during the course of a reaction, it is possible to determine the Fukui functions, which serve to pinpoint the locations of reactivity within a system. As demonstrated in the subsequent equation, chemicals can exist in three distinct environments. The Fukui functions, denoted as *f*^+^(*r*), *f*^−^(*r*), and *f*^*o*^(*r*) are calculated using the formulas [48–50]:

$$f^{-}(r)=q_{k}(N)-q_{k}(N-1)\approx \rho^{HOMO}(r),\;for\;electrophilic\;attack$$

$$f^{+}(r)=q_{k}(N+1)-q_{k}(N\;)\approx \rho^{LUMO}(r),\;for\;nucleophilic\;attack$$

$$f^{0}(r)=\frac{1}{2}[q_{k}(N+1)-q_{k}(N-1)]\approx \frac{1}{2}[\rho^{HOMO}(r)+\rho^{LUMO}(r)],\;for\;Radical\;attack$$

where *q*_*k*_(*N*) is the atomic populations on the *k*_*th*_ atom for the neutral molecule, while *q*_*k*_(*N*+1) and *q*_*k*_(*N*−1) are the atomic population on the *k*_*th*_ atom for its anionic and cationic species, respectively. **S5** and **S6 Tables (see supplementary data)** represent the descriptor values of all compounds **I, V-XI** computed by utilizing use the 6–311++G (d, p) basis group and the wB97XD functionality. In addition, the ability to determine which atomic site in a molecule is electrophilic or nucleophilic is important in addition, Labbe and colleagues [51] proposed an extra Dual descriptor (Δ*f*(r)) The calculation can be determined by using the formula provided below:

$$\Delta f(r)=f^{+}(r)-f^{-}(r),$$

In **S5** and **S6 Tables,** the results showed that the phosphate moiety is the most electrophilic in compounds (**V, VII, VIII**) which is mostly found on the atoms: O39, P40, O41, O42, O43, and C44, but in compounds (**IX-XI)** on atoms O36, P37, O38, O39, O40 and C41. Compound **VI** has the coumarin moiety on the following atoms: O1, C3, C5, C10, and O11. In Deltarazine (**I**), it is mostly found on benzene and benzimidazole moieties atoms: C1, C2, C3, C4, C5, C6, C11, C13, C14, C15, C16, C17, C18 and N19 while the nucleophilic active site in compounds (**V, VII-IX**) The coumarin scaffold contains a functional group at a specific position O1, C2, C3, C4, C6, C8 O11 and O18 (or 17) concentrated. In case **VI** is on the selective site of the coumarin scaffold on the atoms: C2, C4, C6, C8, C9 and C17. In **X-XI**, the selective site of the benzene derivative moiety is on the atoms: C1, C4, C6, C13, C20, N10, O14, O15, O21 and N22. Finally, in Deltarazine (**I**) is mostly found on right arm C61-phenyl and benzimidazole moieties atoms: N60, C61, N62, N64, C66, C67, C68, C73, C74, C75, C76, C77 and C78. Similarly, the identical outcome can be achieved by considering the dual descriptor Δ*f*(r) for both nucleophilic and electrophilic assaults. The high electronegativity of nitrogen and oxygen atoms resulted in a redistribution of electron density, besides the impact of the PO_3_-OCH_3_ anion insertion groups in long alkyl group or derivative of phenyl alternation with benzimidazole groups, that alters these defining characteristics in all substances under investigation **(I, V-XI).**

These results align with the examination of the native population through the determination of Highest Occupied Molecular Orbital (HOMO) and Lowest Unoccupied Molecular Orbital (LUMO). Chattaraj and colleagues introduced the concept of generalized philicity, which involved the utilization of variations in condensed-to-atom Fukui functions., they created a specific measure called philicity, which is associated with a particular site k within a molecule ($f_{k}^{\alpha }$), as shown in the following equation [52]:

$${\omega_{k}^{\alpha }=\omega f}_{k}^{\alpha },$$

In this context, α = +, − and 0 correlate to local philic amounts describing the various types of assaults, including nucleophilic, electrophilic, and radical., respectively. The greatest electrophilic attribute has the largest value $\omega_{k}^{\alpha }$ of, according to the preceding equation. Furthermore, Lee *et al*. [53] introduced various local softness measures to characterize the molecular reactivity, which can be expressed using the following equation:

$${s_{k}^{\alpha }=sf}_{k}^{\alpha },$$

In the equation mentioned above, the variable α denotes the local softness values associated with alpha α, positive (α = +) meaning nucleophilic, negative (α = -) electrophilic, and zero (α = 0) for radical assaults. To obtain a comprehensive study, the CDFT viewpoint was used to determine the local index for both electrophilicity and nucleophilicity, compacted local softness, and percentage electrophilicity/nucleophilicity for each atom in the compounds using the software Multiwfn (version 3.7) [54]. In **S7–S10 Tables**, According to the study’s findings, all the compounds studied displayed both donation and back-donating actions at the active sites, which is consistent with the Fukui functions and frontier orbital theories, these findings are presented in **S5 and S6 Tables.** The results of this study indicate that the compounds under investigation possess multiple active sites, enabling them to interact with the surface of pocket proteins through electron donation. Finally, the previously mentioned local descriptors indicate that the empirical data in this research align with the predicted changes in the effectiveness of the compounds.

#### 2.1.7. Molecular electrostatic potential (ESP)

The utilization of the electrostatic potential (ESP) distribution on molecular surfaces has emerged as a highly efficient method for the identification, analysis, and comprehension of patterns and phenomena [55,56]. In terms of electron density, this can be used to define a molecule’s charge distribution, identify charged regions, and determine which locations are most likely to exhibit bonds of hydrogen, electrophilic, and the nucleophilic features [57]. Electrostatic potentials (ESPs) play a crucial role in the prediction and comprehension of interaction between molecules [56]. It is crucial to undertake a thorough examination of their electrostatic potential (ESPs) in order to fully comprehend the crucial interactions between the synthesized compounds and biological activity. The electrostatic potential (ESP), represented by V(r) in atomic units, is a measure of the electrostatic energy that would be exerted on a positive unit test charge located at a specific point (x, y, z) in the vicinity of the molecule. The ESP can be characterized as the energy of interaction between a proton located at a certain distance, r, and the electrical charge generated by the electrons and nuclei. In this context, negative ESPs are indicative of attractive interactions, while positive ESPs signify repulsion interactions. The electrostatic potential (ESP) distribution can be expressed using the following equation:

$$V(r)=\sum_{A}^{nuclei}\frac{Z_{A}}{\left|R_{A}-r\right|}-\int \frac{\rho (r^{\prime })}{\left|r-r^{\prime }\right)}dr^{\prime },$$

in atomic units, the charge and position of nucleus A are denoted as ZA and RA, respectively. The electron density at position r’ is represented by *ρ* (r’).

**Fig 4:**
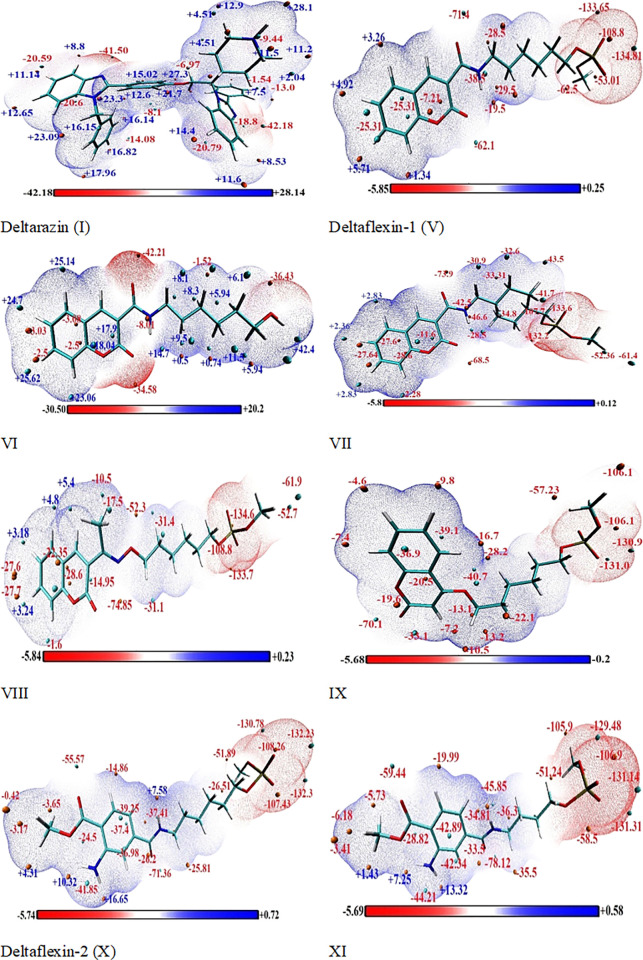
MEP surfaces of selected potential eight target compounds (I, V-XI) utilizing the level of computation wb97xd/6-311++g(d,p).

The active forms of target compounds are represented by surfaces that display their electrostatic potential, **Fig 4** displays the presentation of **I, V-XI.** The Multiwfn package’s module for analyzing the molecular surface quantitatively allows us to divide the overall van der Waals surface into multiple fragments. This functionality allows us to examine the attributes of the ESP distribution.

Regarding compounds **(V, VII-VII)** The ESP value on the surface is significantly negative that localized on phosphate group and oxygen of lactam ring of coumarin nucleus at the O39, P40, O41, O42, O43, C44, O1 and O11 (-133.6, -108.8, - 134.8, -53.01, -62.5, -62.1, -25.31 and -7.21 Kcal/mol), respectively, but sequence in compounds (**IX-XI)** localized on phosphate group with oxygen and nitrogen of phenyl derivatives -COOMe and NH_2_ electron withdrawing groups which decrease electron cloud on atoms O36, P37, O38, O39, O40, C41, O1 and O11 (-129.5, -106.9, - 131.14, -58.5, -35.5, -78.12, —33.5 and -48.8 Kcal/mol), respectively. In compound **VI** is mainly localized on the selective site of the coumarin moiety on the atoms: O1, C3, C5, C10, O11 and O39 (-34.58, -23.06, -3.08, -8.01 and -36.43 Kcal/mol), respectively. Deltarazine (**I**) is mostly found on benzene and benzimidazole moieties atoms specifically N and O at: C1, C3, C4, C11, N12, C13, N19, O39, N60, N62 and N47 (-8.1, -14.08, -20.6, -41.5, -20.79, -42.18 and -9.44 Kcal/mol), in the order mentioned. The carbon atom hosting the proton in the derivatives exhibits the highest energy state on the surfaces of the derivatives’ derivatives (I, V-XI). The energy values range from +28.14 to +5.2 Kcal/mol for the derivatives. This suggests that the primary mode of interaction between the active forms of eight target compounds and their target receptors, to suppress oncogenic k-Ras, will be either electrostatic or hydrogen bonding. The ESP values demonstrate the capacity to form hydrogen bonds and undergo transfer charges between molecules. These results are consistent with assessments of the NBO population as well as local reactivity characteristics.

#### 2.1.8. Structural Activity Relationships (SAR)

In this research, the physicochemical characteristics of the targeting compounds (**I**, **V-XI)** were determined and recorded in **Table 4**. These properties include molar volume (V), molar refractivity (MR), surface area grid (SAG), hydration energy (HE), and polarizability (Pol). The calculations were performed *via* HyperChem software (v8.0.7). molecular polarizability refers to the ability of a system of electronics to effectively respond and adapt to the influence of an outside electrical field generated by light. The importance of molecular polarizability lies in its fundamental contribution to the simulation of various compound properties and biological activities [36]. The volume of a molecule plays a crucial role in affecting different physiological functions like the penetration of the blood-brain barrier and the absorption in the intestines. Additionally, it is the primary factor that governs the polarizability of molecules. Therefore, it is imperative to incorporate molecular volume as a parameter in quantitative structure-activity relationship (QSAR) studies to model molecular properties and biological activities accurately. Another SAR characteristic that can be considered is molar refractivity (MR), which is a steric property that relies on the spatial configuration of benzene moiety in the molecules being analyzed. The spatial configuration holds great importance as it plays a critical role in comprehending the way medication molecules engage with receptors. The dispersion of London force, which plays a crucial role in the relationship among drug molecules and receptors, is an additional contributing factor to the determination of molar refractivity, in addition to its dependence on molecular volume.

**Table 4:** Physico-chemical properties analysis and QSAR properties of selected potential eight target compounds (I, V-XI) towards PDEδ inhibitors for repression of oncogenic k-Ras.

Parameters	Polarizability^1^	Refractivity^1^	Vol ^1^	Surface area (Grid)^2^	HE^3^	Log P	MW^4^
Deltarazin (I)	74.09	182.71	1687.64	932.53	-8.19	8.18	603.77
Deltaflexin-1 (V)	35.49	92.57	1070.45	657.34	-10.89	3.63	382.33
VI	31.08	77.84	889.28	551.63	-9.86	1.42	289.33
VII	38.39	99.71	1104.51	675.58	-9.82	3.93	408.37
VIII	37.46	97.8	1140.83	699.77	-11.1	4.54	396.36
IX	32.86	85.56	996.99	609.11	-9.86	4.29	355.3
Deltaflexin-2 (X)	34.71	95.5	1109.49	665.85	-11.57	3.14	387.35
XI	31.88	86.3	1001.82	611.64	-12.3	2.34	359.3

^1^ Data in (A^3^) unit

^2^ Data in (A^2^) unit

^3^ Data in (Kcal/mol) unit

^4^ Data in (DA) unit.

According to the findings shown in **Table 4**, it is evident that the dimensions (volume) and molecular weight (MW) of the suggested compounds exhibit a positive correlation with molecular refractivity, polarizability data, and surface area grid. Deltarazin (**I**) has the highest volume value (1687.64 Å^3^), refractivity (182.71 Å^3^), maximum polarizability value (74.09 Å^3^), surface area grid (932.53 Å^3^) also, It possesses the greatest molecular weight (MW) among all substances, measuring 603.77 atomic mass units (amu). In contrast, compound VI exhibits lower values for all four parameters: polarizability, refractivity, molecular volume, molecular weight and surface area grid, with corresponding values of (31.08 Å3, 77.84 Å3, 889.28 Å3, 289.33 amu 551.63 Å2,). Other chemicals’ gradients within **Table 4** get progressively smaller as **VII>VIII> Deltaflexin-2 (X)> Deltaflexin-1 (V)>IX** The identical pattern is observed across all eight target compounds.

**Table 4** showed that the values of hydrophobicity have been rising, which causes the energy required for hydrolysis to decrease. The energy of water intake controls how stable different molecule structures are in water solutions [58,59]. The increase or reduction in hydrogen bonding (acceptors and donors) influences how the energy value of hydration changes. The exact hydration energy data (**Table 4)** were organized as follows: **Deltarazin** (**I**)<**VII<IX<VI< Deltaflexin-1 (V)<VIII< Deltaflexin-2 (X)<XI** with values of (-8.19, - 9.82, -9.86, -9.86, -10.89, -11.1, -11.57, -12.3 Kcal/mol), in the order mentioned and are comprised of acceptors and donors for hydrogen bonding.

Numerous ADME characteristics are impacted by the lipophilic nature of compounds. Log P is a parameter that quantifies the distribution of medicinal compounds among the watery environment surrounding the cell membrane and the lipid composition of the membrane itself. This finding suggests that compounds with reduced Log P values exhibit higher polarity and lower permeability through lipid bilayers, whereas molecules with greater Log P values are less polar and exhibit reduced solubility in water [60,61]. Consequently, except for **Deltarazin (I)** which possesses a logarithmic partition coefficient (log P) of 8.18, all other compounds exhibit favorable solubility in aqueous environments. In addition, the values of *Log P* for target compounds **Deltarazin**(**I**)>**VIII>IX>VII>Deltaflexin-1 (V)>VIII> Deltaflexin-2 (X)>XI>VI** are in the ideal range (0 < *Log P* < 5) [62]. These hybrid compounds exhibit the most effective biological activity and are easily absorbed when taken orally. The **Deltarazin (I)** compound requires a carrier for drug delivery to be deposited onto a nanomaterial with specific properties that will improve its oral bioavailability.

#### 2.1.9. The structure-activity relationship (SAR) for the eight derivatives studied

The structure-activity relationship of eight compounds, as determined by biological assays, indicated that the standard compound Deltarazine (I) exhibited the highest activity may be due to the presence of the bis-benzimidazole scaffold in addition to the morpholine moiety. Deltaflexin-1 (V) also demonstrated significant activity attributed to the coumarin scaffold, while its derivative, deltaflexin-2 (X), showed increased activity (from 4.87 ± 0.03 to 3.94 ± 0.03μm) upon replacement of the coumarin scaffold with the etesteramine ring. This change may act as isosters of the secondary amine pyridazine scaffold in Deltasonamides (III) compounds. Conversely, substituting the hexyl group in deltaflexin-1 with a rigid cyclohexyl moiety led to decreased activity (reaching 6.7 ± 0.1μM). Additionally, the absence of a phosphate group significantly reduced the activity of compound VI to 18.9 ± 0.2μM. Furthermore, reducing the number of aliphatic groups from hexyl to pentyl and changing the leaving group resulted in decreased activity of compound XI. Finally, both derivatives of coumarin alkoxy (IX) and aminoxy (VIII) exhibited lower biological activity compared to the previous compounds.

### 2.2. In silico physicochemical properties and pharmacokinetic parameters

The compound features were generated using the Swiss ADME online method. Most of the compounds showed a high to moderate solubility profile, different lipophilicity revealed by the different Wlogp values, no blood-brain barrier permeability and high gastrointestinal (GI) absorption except for compound **X** which showed low GI absorption. The chemicals’ similarity to drugs was made clear by their zero violation of Lipinski’s rule of five, whereas the medicinal chemistry friendliness of the compounds was confirmed by their zero PAINS alerts (pain-assay interference structural alerts). To summarize, the tested compounds could demonstrate promising lead like properties, **Table 5**.

**Table 5:** Compound physicochemical and ADME characterization.

Properties	I	V	VI	VII	VIII	IX	X	XI
#Heavy atoms	46	26	21	28	27	24	26	24
#Rotatable bonds	9	11	8	8	11	10	13	11
#H-bond acceptors	4	7	4	7	8	7	7	7
#H-bond donors	1	2	2	2	1	1	3	3
MR	197.64	96.32	80.68	103.82	102.67	89.81	95.96	86.35
TPSA	54.68	124.88	79.54	124.88	117.37	105.01	146.99	146.99
WLOGP	6.15	2.85	2.08	3.09	3.86	3.50	2.12	1.34
SOL Class	Poorly Soluble	Soluble	Soluble	Soluble	Soluble	Soluble	Soluble	Very Soluble
GI absorption	High	High	High	High	High	High	Low	Low
BBB permeant	No	No	No	No	No	No	No	No
Lipinski #violations	2	0	0	0	0	0	0	0
Bioavailability Score	0.55	0.56	0.55	0.56	0.56	0.56	0.56	0.56
PAINS #alerts	0	0	0	0	0	0	0	0
Synthetic Accessibility	5.99	3.92	2.85	4.53	4.57	4.21	3.29	3.12

Those findings suggest the selected compounds possess excellent pharmacodynamics and pharmacokinetic properties making them excellent candidates for future drug optimization. Only compounds X and XI would need further optimization through adding hydrophobic substitutions.

### 2.3. Molecular docking

**Fig 5:**
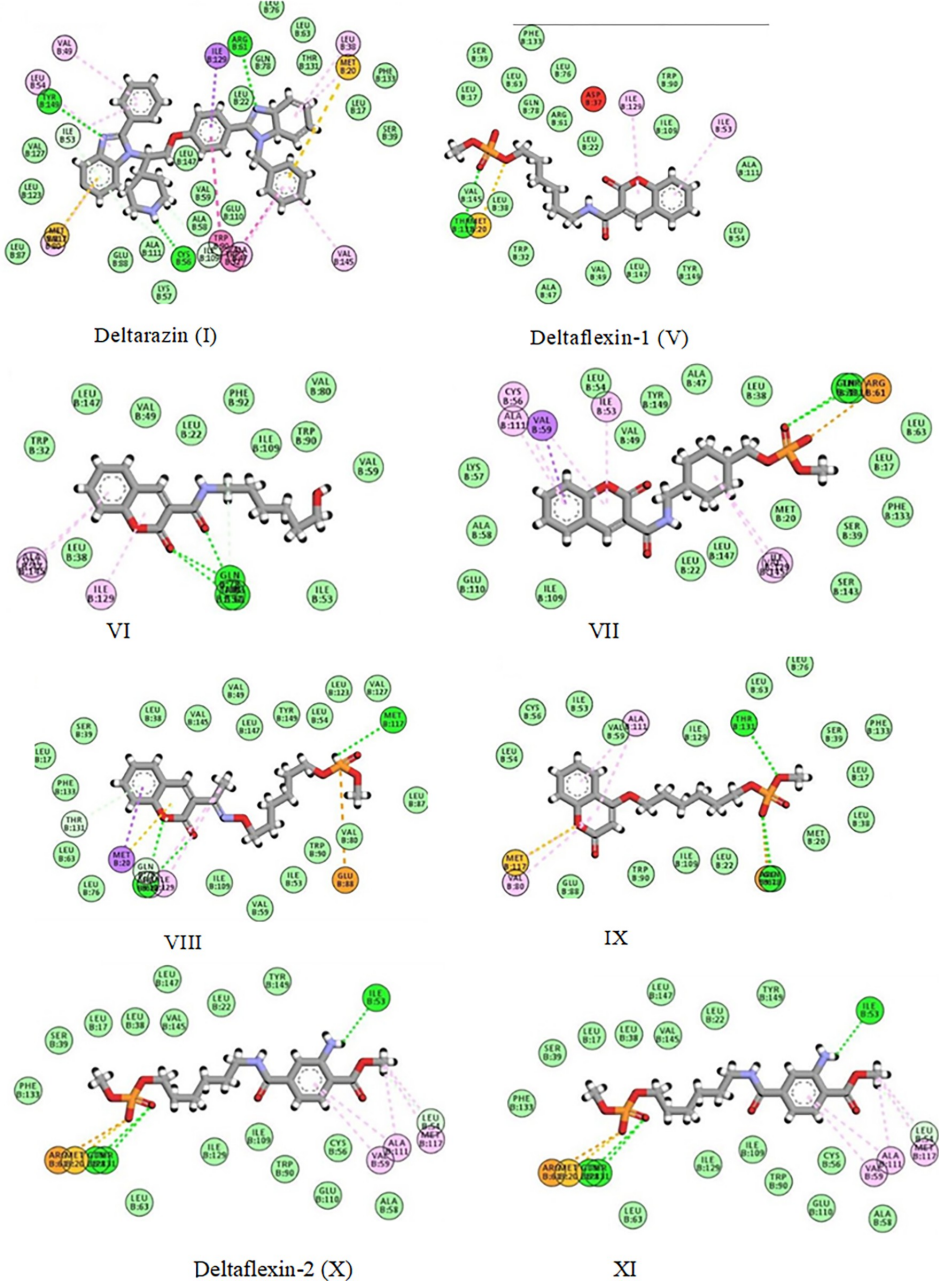
Molecular docking of the target compounds.

In this section, we aimed not only to clarify the potential ways in which the reported compounds can bind but also to compare them on the basis of docking scores, further comparing the retrieved docking scores for all the compounds with the experimental biological results against the enzyme. The docking results demonstrated a preferred binding mode for all the docked compounds, indicated by the negative binding energy score achieved by the eight compounds. More interestingly, the docking results were perfectly matched with the experimental biological assay, as demonstrated in **Table 6**. The 2D interaction diagrams for the eight compounds with the target are represented in **Fig 5.** Three hydrogen bonds were observed in **Deltarazine** interaction with the receptor through its nitrogen atoms in the two benzimidazole rings and piperidine ring. Furthermore, its aromatic π system tangled in either π- π interaction with TRP90 and TRP32, sulfur-π interaction with MET20 and MET117 or π-alkyl interactions. The phosphate groups in target compounds (**V**, **VII-XI**) involved in hydrogen bonds and either sulfur-π interaction with MET20 or electrostatic attraction with ARG61 or GLU88. In addition, the coumarin ring and its carbonyl side chain in **VI** formed hydrogen bonds through its oxygen atoms and the ring of **VIII** participated in hydrogen bond interactions. Moreover, various hydrophobic interactions were seen and presented in **Fig 5**.

**Table 6:** The binding energy score and biological activity of the eight compounds.

Compound	Biological activity	Score (Kcal/mol)	Residues involved in hydrogen and hydrophobic interactions
Deltarazin (I)	1.9 ± 0.1	-10.67	Cys56, Arg61, Tyr149, Met20, Ala47, Tyr52, Met117, Leu38, Ile53, Leu54, Val49, Leu38, Ile129, and Val145
Deltaflexin-1 (V)	4.87 ± 0.03	-10.1	Thr131, Met20, Ile53, Ile129
VI	18.9 ± 0.2	-9.77	Gln78, Arg61, Thr131, Ala47and Val145
VII	6.7 ± 0.1	-9.98	Gln78, Arg61, Thr131, Ile129, Ile53, Cys56, Val59, Ala111 and Val145
VIII	-	-9.3	Gln78, Arg61, Thr131, Met20, Ile129, Met117, Ala111 and Glu88
IX	-	-9.4	Gln78, Arg61, Thr131, Ala111, Met117 and Val80
Deltaflexin-2 (X)	3.94 ± 0.03	-10.2	Arg61, Met20, Met117, Ile53, Leu54, Thr131, Gln78, Val59, and Ala111
XI	-	-8.91	Arg61, Thr131, Ala111, Val59, Ile53, Leu54, and Met117

### 2.4. Molecular dynamics

**Fig 6:**
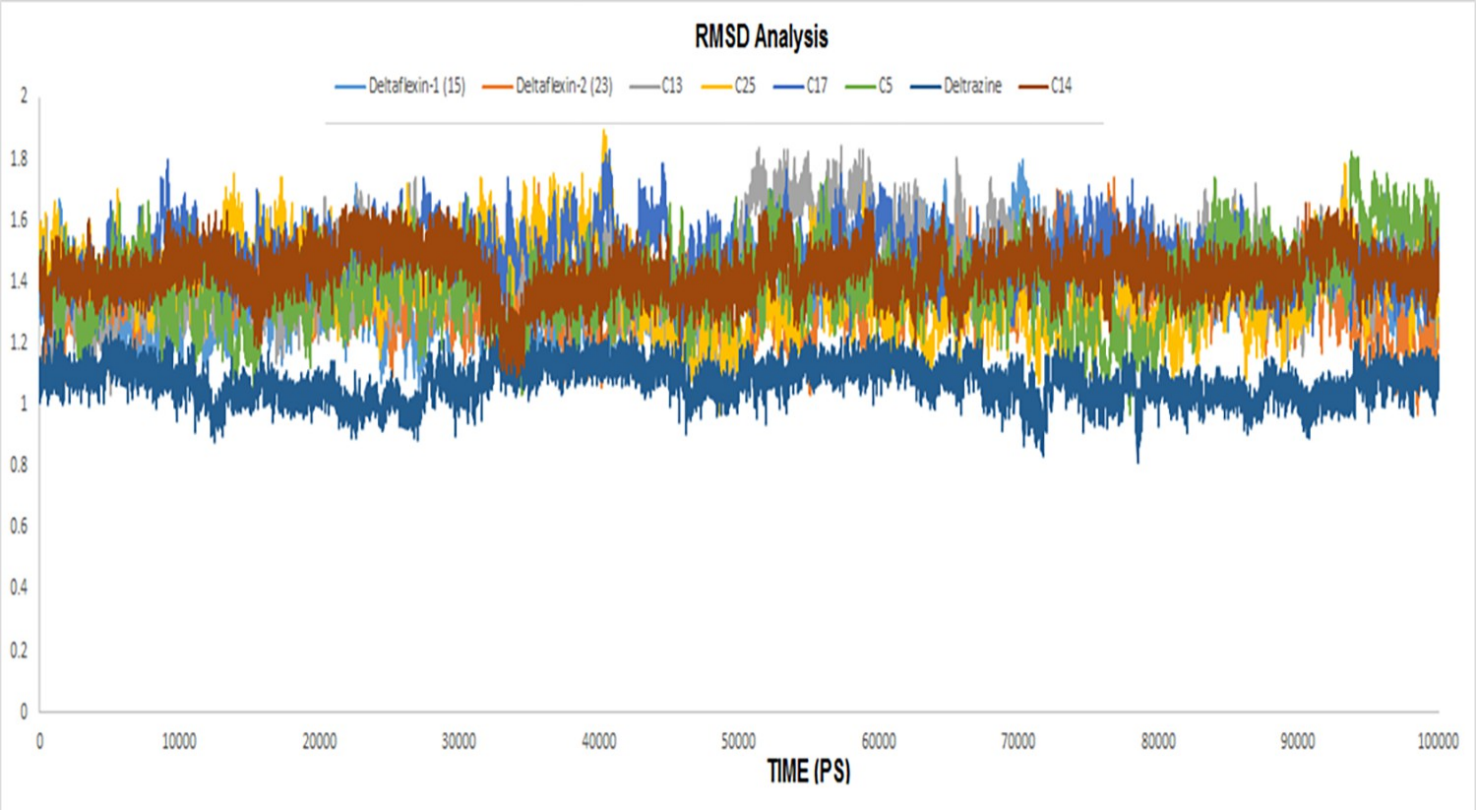
The RMSD analysis for the entire residues of the eight compounds in complex with their target.

The molecular dynamics technique is of important value in many drug discovery studies, including but not limited to the discovery of novel cures for emerging diseases, the characterization of the behavioral nature of macromolecules, the interpretation of amino acid mutations on drug resistance cases and the predictive estimation of the strength of binding between the drugs and their targets [63,64]. The last one was our primary goal from the molecular dynamic simulations in addition to the validation of the docking results retrieved from the docking process. The score for docking is determined using only one conformation, while the molecular dynamics calculations rely on millions of ligand-receptor conformations. Accordingly, eight molecular dynamic simulations were conducted for each compound bound to its target. The results of the molecular dynamics revealed a stable binding mode for all the ligands with their corresponding target as highlighted by the low RMSD for each complex (**Fig 6**).

Also, the RMSD showed fluctuations less than 1 Å from initial conformation that was retrieved from the docking. **Table 7** summarizes the RMSD values of the eight complexes.

**Table 7:** The RMSD of the eight complexes at their maximum fluctuation.

Complex	RMSD
**Deltarazin (I)**	1.24
**Deltaflexin-1 (V)**	1.79
**VI**	1.65
**VII**	1.83
**VIII**	1.82
**IX**	1.84
**Deltaflexin-2 (X)**	1.73
**XI**	1.82

**Fig 7:**
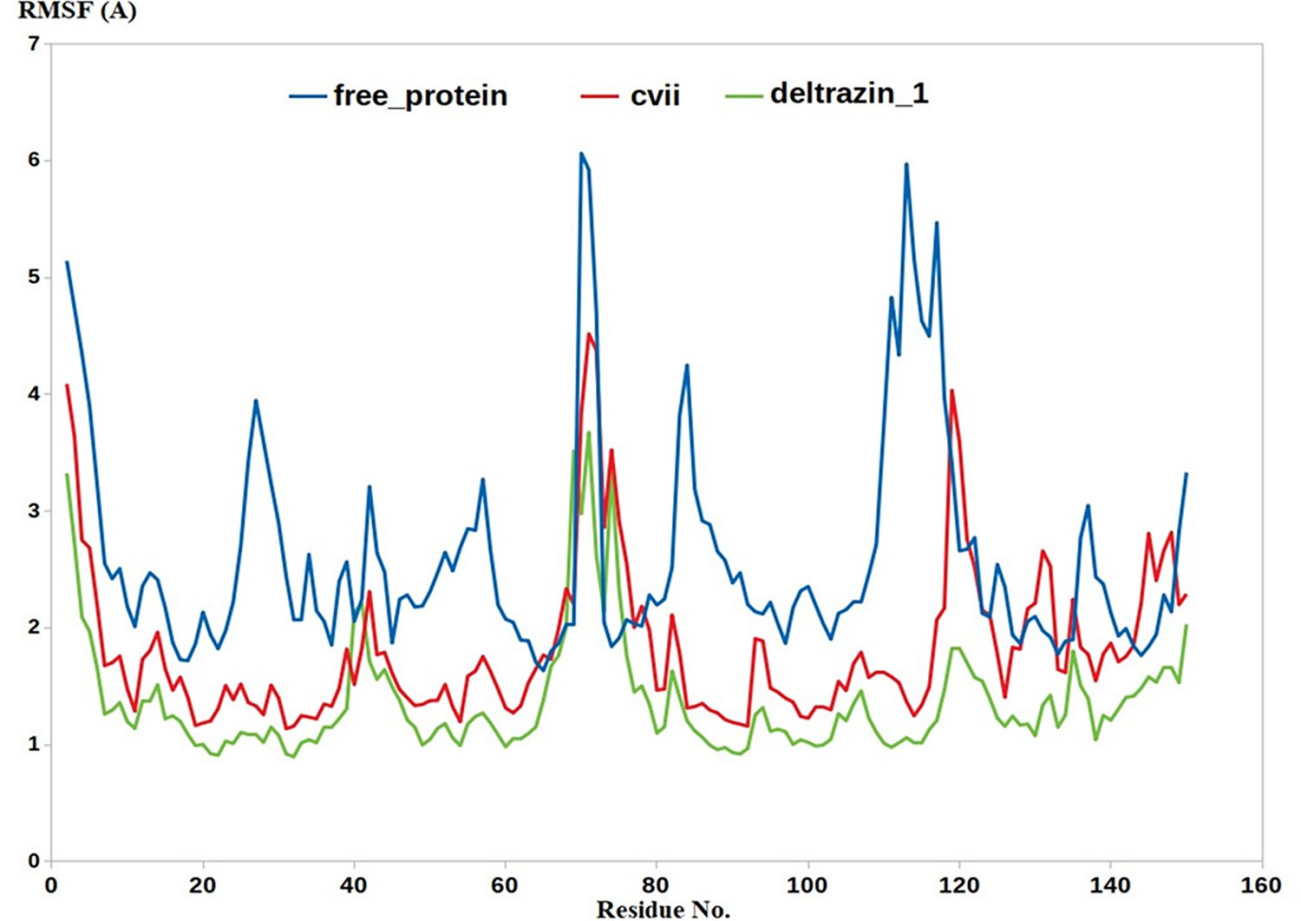
The RMSF calculation through the entire MD.

**Fig 8:**
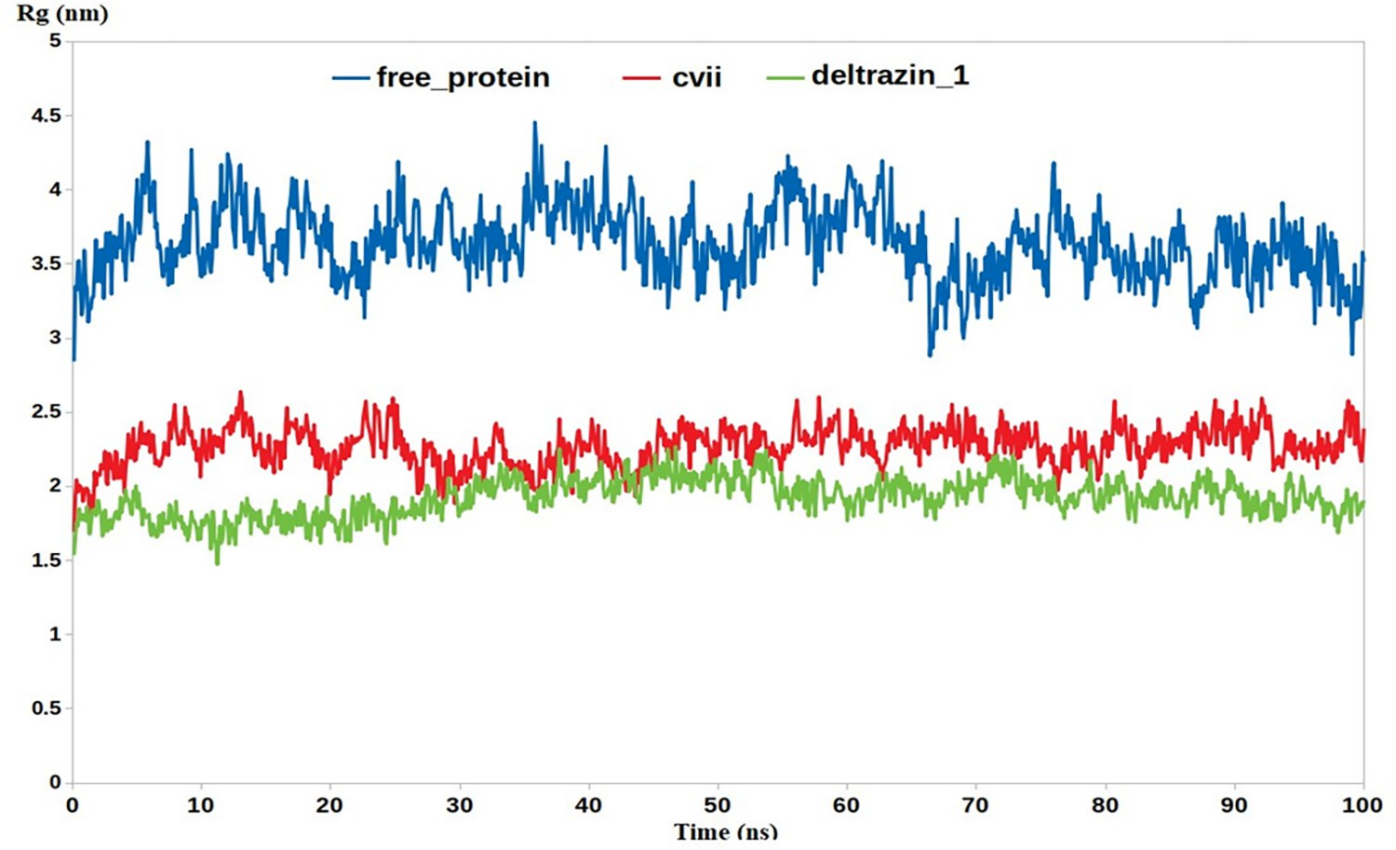
The RG calculation through the entire MD.

Further parameters were also computed to provide extra validation, this includes RMSF and radius of gyration (RG). The results demonstrated excellent alignment with all the insilico results, where the apo protein should high fluctuations in both RMSF and RG. In contrast, **Deltarazin (I) and to lesser extent compound VII showed excellent ability to lower the fluctuation of RG and RMSF as compared with the apo protein, Figs 7 & 8.**

### 2.5. MM-PBSA calculations

The binding free energy of the PDEδ receptor and its interaction with eight compounds was determined through the utilization of the MM-PBSA binding free energy procedure. This approach computes the binding free energy by analyzing multiple molecular dynamics conformations stored in the trajectory file [65,66]. Therefore, the technique can be more reliable when compared to the docking study, which involves only a single conformation-based estimation. The sum of a ligand-protein complex’s free energy is estimated by adding together the free energy of solvation and the potential energy from Vacuum molecular mechanics. The solvation free energy consists of two components: the polar electrostatic solvation energy and the nonpolar non-electrostatic solvation energy, which is determined using the SASA model. **Table 8** presents an overview of all MM-PBSA outcomes and energy forms. Generally, the calculated results showed the capability of each compound to form stable complexes with PDEδ as evidenced by all eight complexes having negative favorable binding free energy. More importantly, the MD, MM-PBSA and docking results support each other, revealing the power of computational approaches and calculations in the prediction and interpretation of experimental results.

**Table 8:** MM-PBSA calculations of the binding free energy for the eight complexes.

Complex^1^	ΔE binding	ΔE Electrostatic	ΔE Vander Waal	ΔE polar solvation	SASA
**Deltarazin (I)**	-350.58± 18.53	-139.05 ± 14.67	-284.13 ± 23.84	101.01 ± 14.09	-28.41± 3.17
**Deltaflexin-1 (V)**	-332.92± 18.33	-134.72 ± 16.11	-273.94 ± 22.82	99.35± 13.06	-23.61± 2.84
**VI**	-313.19± 16.74	-129.08 ± 15.07	-247.85 ± 21.52	83.91± 12.14	-20.17± 2.04
**VII**	-317.25± 16.27	-128.49 ± 14.80	-255.17 ± 20.14	85.77 ± 11.85	-19.36± 1.99
**VIII**	-300.92± 16.40	-123.53 ± 13.95	-240.06 ± 22.45	79.98± 10.87	-17.31± 2.13
**IX**	-285.02± 15.86	-118.93 ± 13.75	-232.86 ± 19.98	85.19± 11.76	-18.42± 1.87
**Deltaflexin-2 (X)**	-339.03± 18.10	-132.13 ± 15.27	-280.28 ± 24.01	98.77 ± 12.78	-25.39± 2.25
**XI**	-279.27± 15.12	-120.84± 13.62	-222.97 ± 19.43	80.26± 10.74	-15.72± 1.66

^1^ Energies in (kj/mol) unit.

## 3. Materials and methods

### 3.1. DFT computational details

During this study, the molecular modeling calculations of k-Ras inhibitors of eight potential target derivatives were carried out using software of Gaussian 09W [67]. The molecular structure of a specific hybrid coumarin derivative (VI) was completely optimized using three different approaches. These included Hartree-Forck (HF) [1], density functional theory with the Becke’s three parameter exchange functional and the gradient corrected functional of Lee, Yang and Parr (DFT/B3LYP) [2–5], and long-range corrected (LC)-DFT (wB97XD) [6], utilizing two distinct basis sets: 6–311++G (d, p) and 6–311+G (d) [7].

The target derivatives’ molecular structures were subjected to geometric optimization using DFT with the wB97XDage functional, which incorporates long-range corrections [68] through 6–311++G (d, p) basis set [69]. Symmetry constraints were not employed during the process of geometry optimization [70,71]. To enhance the precision, uniformity, adaptability, and overall effectiveness of Grimme’s method, D2 dispersion that incorporates empirical dispersion was employed [67–72]. The compound’s respective vibrational frequencies were obtained using the previous theory, and the molecular structure of the target compounds represents actual low-energy points on the surface of potential energy. To determine the specific location of reactivity within the molecules, the wB97XD functional method was utilized to analyze descriptors of reactivity and stability at the molecular level. Based on the Fukui function and the Twin descriptor, we constructed the local reactivity descriptor [44–46,48–51].

Additionally, the Multiwfn v3.7 software program utilized to acquire the descriptors of quantum chemistry based on conceptual density functional theory (CDFT) [54]. Descriptors of quantum chemistry were employed for producing electrostatic potential (ESP) of the target compounds and viewed by the Visual Molecular Dynamics package (VMD 1.9 program) [73]. Natural Bond Orbital (NBO) analysis was obtained via NBO 3.1 in the Gaussian 09W program. The optimized structure and molecular orbitals were visualized using the program of GaussView (v6.1) [74] and ChemCraft (v1.6) software packages [59]. The SAR characteristics of the target molecules were assessed through utilizing the features of quantative structure-activity relationship integrated into the HyperChem software (version 8.0.7) [75].

### 3.2. In silico physicochemical properties and pharmacokinetic parameters

The *SwissADME* web tool available from the Swiss Institute of Bioinformatics (SIB) was utilized to generate the physicochemical properties and pharmacokinetic parameters of the tested molecules [76,77].

### 3.3. Molecular docking

The MOE2019 software was used to perform all docking studies. The crystallographic structure of PDEδ was acquired from the Protein Data Bank with the PDB ID **4JVb** [78], and the binding site was defined based on the position of the ligand that has formed a co-crystal. The typical workflow for molecular modeling protocol was applied, beginning with preparing the receptor by adding hydrogens, adjusting charges, and minimizing energy under the AMBER12: EHT10 force field [79]. The eight compounds and co-crystalized ligand were prepared in a single file with *.mdb extension as required by MOE 2019. The docking was performed in two stages. Firstly, the docking protocol was initially validated as the ligand that has been co-crystalized, was retrieved and re-docked to its corresponding active site. This process led to an RMSD of 0.94 Å between the co-crystalized pose and the retrieved docked pose, indicating a valid protocol for docking. In the second phase, the eight compounds were inserted into the target’s binding site through docking. Discovery Studio visualizer 2019 used the docking data to create a 2D interaction graph [80].

### 3.4. Molecular dynamics

The molecular dynamics (MD) simulation experiments in this study were performed following the established protocols outlined in the published literature by our previous studies [81]. The standard procedure of running GROMACS 2020.4 software, known as GROningen MAchine for Simulations of chemistry, was followed to perform eight molecular dynamic simulations of ligand-protein complexes [82]. The topologies of the eight ligands were generated and joined with the receptor topology to yield eight complexes. The solvation of the eight systems was accomplished by employing the water model known as Single Point Charge (SPC) before neutralizing them by adding suitable counter-ions. The steepest descent minimization algorithm, this technique was used to reduce the energy of all systems. This process was carried out for a maximum of 50,000 steps, ensuring that the force remained below 10.0 kJ/mol. The GROMOS96 43a1 force field was utilized for this purpose [83]. After minimizations of energy, all the systems were equilibrated for 2ns of NVT ensemble simulation (constant number of particles, volume, and temperature (310 K)). An additional equilibration step was conducted, employing the NPT ensemble, which preserves a constant number of particles, temperature and pressure, within a duration of 8 nanoseconds while the long-range electrostatic was upheld [84]. Finally, balanced systems reached a production stage of 100 nanoseconds. The trajectories were monitored at regular intervals of 10 picoseconds to record the structural coordinates. Using production trajectories, RMSDs were calculated for all system residues.

## 3.5. MM-PBSA calculation

To determine the binding free energy[85], a conventional equation was employed in the following manner:

$$\Delta G_{\left(Binding\right)}=G_{\left(Complex\right)}‐G_{\left(Receptor\right)}‐G_{\left(Ligand\right)},$$

where, G_(Complex)_, G_(Receptor)_ and G_(Ligand)_ are the total free energy of the protein−ligand complex, free enzyme and ligand in solvent, respectively (**supplementary Information**).

## 4. Conclusions

In this study, some computational techniques were used to detect the bioactivity effect of k-Ras inhibitors on the prenyl-binding pocket of protein PDEδ. The equilibrium geometries and vibrational frequencies that impact the description of the thermochemistry and subsequent calculation of the optical properties of eight k-Ras inhibitor target compounds were determined using DFT functional. According to the analysis, the results showed that the calculated binding free energies using MM-PBSA were consistent with the experimental data. The RMSD depths for Deltrazin/ PDEδ and Deltaflexin/ PDEδ are quite similar, but other inhibitors are different. The phosphate group had a crucial role in the inhibitors activity and forms stable and strong H-bond interaction with THR131, which causes the hydrophobic to be induced scaffold of coumarin close to the other residue, such as ILE 129, leading to the formation of strong hydrophobic interactions. Compared with Deltrazine there is H-bond interaction with ARG61 and TYR149 and also some hydrophobic interaction. To conclusion, these results outspread the understanding of the dissociation mechanism of PDEδ inhibitors to provide further information for the design and synthesis of improved novel KRAS-PDEδ interactions.

## Supporting information

S1 Fig Redocking study showing excellent superimposition between the co-crystalized and the retrieved docking pose. (TIF)

S1 Table The selected bond length (Å), bond angles and dihedral angles, (degree) of the selected potential target compounds (V-IX) of Coumarin derivatives using wb97xd/6-311++g(d,p) level of theory verses the X-ray crystal structure data (CCDC: 697425) of 3-((2-Oxo-2H-chromen-3-yl)carbonyl)pyridinium hydrogen squarate. (DOCX)

S2 Table Mean absolute errors computed for selected bond lengths (Ao) and angles (degree) of the selected potential target compounds (V-IX) of Coumarin derivative verses 3-((2-Oxo-2H-chromen-3-yl)carbonyl)pyridinium hydrogen squarate calculated at long-range corrections wb97xd/6-311++g(d,p) level of theory. The X-ray crystal structure data (CCDC: 697425). (DOCX)

S3 Table The selected bond length (Å), bond angles and dihedral angles, (degree) of selected potential target compounds Deltarazin and Deltaflexin derivatives (I, X- XI) using wb97xd/6-311++g(d,p) level of theory; S4 Table: Natural charge of selected atoms of selected potential eight target compounds (I,V-XI) by using wb97xd/6-311++g(d,p) level of theory. (DOCX)

S4 Table Natural charge of selected atoms of selected potential eight target compounds (I,V-XI) by using wb97xd/6-311++g(d,p) level of theory. (DOCX)

S5 Table Values of the Fukui functions and Dual descriptor of selected potential target compounds (V-XI) using wb97xd/6-311++g(d,p) level of theory. (DOCX)

S6 Table Values of the Fukui functions and Dual descriptor of selected potential target compounds (I, X-XI) using wb97xd/6-311++g(d,p) level of theory. (DOCX)

S7 Table Values of the Condensed local Softnesses (Hartree*e) of selected potential target compounds (V-IX) by using wb97xd/6-311++g(d,p) level of theory from CDFT point of view. (DOCX)

S8 Table Values of the Condensed local Softnesses (Hartree*e) of selected potential target compounds (I, X, XI) by using wb97xd/6-311++g(d,p) level of theory from CDFT point of view. (DOCX)

S9 Table Values of the Condensed local electrophilicity (ElP)/nucleophilicity (NuP) index (e*eV) of selected potential target compounds (V-IX) by using wb97xd/6-311++g(d,p) level of theory from CDFT point of view. (DOCX)

S10 Table Values of the Condensed local electrophilicity (ElP)/nucleophilicity (NuP) index (e*eV) of selected potential target compounds (I, X-XI) by using wb97xd/6-311++g(d,p) level of theory from CDFT point of view. (DOCX)
