# Supplementary figures and images for: DFT and molecular simulation validation of the binding activity of PDEδ inhibitors for repression of oncogenic k-Ras
Source: PLoS One. 2024 Mar 8;19(3):e0300035. doi: 10.1371/journal.pone.0300035 (PMC10923412; doi:10.1371/journal.pone.0300035)

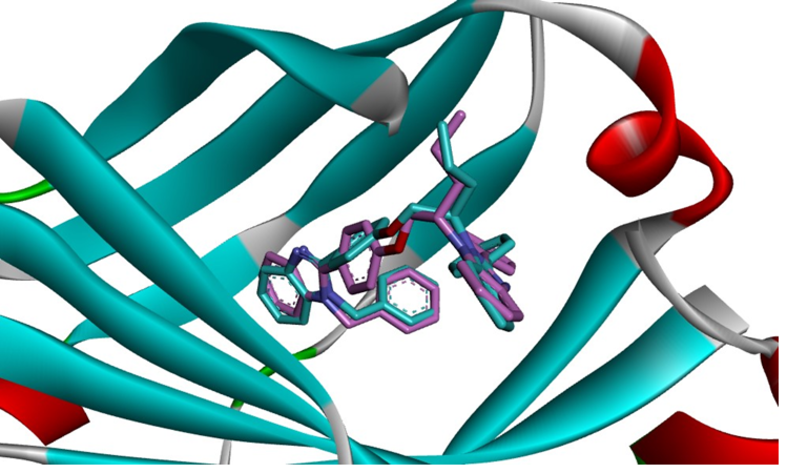

Supplement: S1 Fig — (TIF) [file pone.0300035.s001.tif]
