# Supplementary material for: DFT and molecular simulation validation of the binding activity of PDEδ inhibitors for repression of oncogenic k-Ras
Source: PLoS One. 2024 Mar 8;19(3):e0300035. doi: 10.1371/journal.pone.0300035 (PMC10923412; doi:10.1371/journal.pone.0300035)
Supplement: S1 Table — (DOCX) [file pone.0300035.s002.docx]

**Table S1.** The selected bond length (Å), bond angles and dihedral angles, (degree) of the selected potential target compounds (**V-IX)** of Coumarin derivatives using wb97xd/6-311++g(d,p) level of theory verses the X-ray crystal structure data (**CCDC: 697425**) [24].of **3-((2-Oxo-2H-chromen-3-yl)carbonyl)pyridinium hydrogen squarate**

|  | **Experimental** | V | VI | VII | VIII | IX | |
| --- | --- | --- | --- | --- | --- | --- | --- |
| R(O1,C2) | 1.438 | 1.374 | 1.370 | 1.375 | 1.385 | 1.384 | |
| R(O1,C10) | 1.458 | 1.356 | 1.357 | 1.355 | 1.354 | 1.356 | |
| R(C2,C3) | 1.324 | 1.467 | 1.466 | 1.468 | 1.481 | 1.447 | |
| R(C2,O11) | 1.328 | 1.204 | 1.206 | 1.203 | 1.192 | 1.201 | |
| R(C3,C4) | 1.379 | 1.348 | 1.350 | 1.348 | 1.349 | 1.356 | |
| R(C3,C17) | 1.435 | 1.516 | 1.513 | 1.516 | 1.481 | 1.079 | |
| R(C4,C5) | 1.397 | 1.435 | 1.433 | 1.435 | 1.440 | 1.454 | |
| R(C5,C6) | 1.425 | 1.402 | 1.403 | 1.402 | 1.401 | 1.399 | |
| R(C5,C10) | 1.330 | 1.396 | 1.396 | 1.396 | 1.395 | 1.395 | |
| R(C6,C7) | 1.313 | 1.381 | 1.380 | 1.380 | 1.382 | 1.382 | |
| R(C7,C8) | 1.367 | 1.399 | 1.400 | 1.399 | 1.398 | 1.397 | |
| R(C8,C9) | 1.401 | 1.383 | 1.383 | 1.383 | 1.384 | 1.383 | |
| R(C9,C10) | 1.294 | 1.391 | 1.391 | 1.391 | 1.391 | 1.392 | |
| R(C17,O18) | 1.254 | 1.223 | 1.222 | 1.224 |  |  | |
| R(C17,N19) |  | 1.341 | 1.344 | 1.339 | 1.278 |  | |
| R(N19,C21) |  | 1.455 | 1.451 | 1.454 |  |  | |
| R(C21,C24) |  | 1.522 | 1.522 | 1.525 | 1.514 | 1.527 | |
| R(C36,O39) |  | 1.408 | 1.419 | 1.408 | 1.408 |  | |
| R(O39,P40) |  | 1.650 |  | 1.650 | 1.650 | R(O36,P37) | 1.670 |
| R(P40,O41) |  | 1.488 |  | 1.487 | 1.488 | R(P37,O38) | 1.489 |
| R(P40,O43) |  | 1.672 |  | 1.671 | 1.672 | R(P37,O40) | 1.664 |
| R(O43,C44) |  | 1.405 |  | 1.405 | 1.404 | R(O40,C41) | 1.407 |
| A(O1,C2,C3) | 117.81 | 116.73 | 116.80 | 116.70 | 115.94 | 116.98 | |
| A(C3,C2,O11) | 127.55 | 126.71 | 126.53 | 126.73 | 126.85 | 127.73 | |
| A(C4,C3,C17) | 116.57 | 117.11 | 117.24 | 117.12 | 117.95 | 118.26 | |
| A(C3,C4,C5) | 120.78 | 121.80 | 121.75 | 121.82 | 122.00 | 119.71 | |
| A(C4,C5,C6) | 122.39 | 124.01 | 123.97 | 123.98 | 123.99 | 123.41 | |
| A(C4,C5,C10) | 117.18 | 117.32 | 117.33 | 117.35 | 117.35 | 117.33 | |
| A(C7,C6,H13) | 120.87 | 120.74 | 120.81 | 120.82 | 120.53 | 121.00 | |
| A(C8,C9,C10) | 118.43 | 118.72 | 118.65 | 118.75 | 118.95 | 119.19 | |
| A(O1,C10,C5) | 120.89 | 120.96 | 120.89 | 120.94 | 121.00 | 122.13 | |
| A(C5,C10,C9) | 120.38 | 121.51 | 121.55 | 121.50 | 121.39 | 120.78 | |
| A(C3,C17,N19) |  | 116.92 | 116.74 | 116.92 | 116.01 |  | |
| A(O18,C17,N19) |  | 123.81 | 123.49 | 123.89 | 123.47 |  | |
| A(C17,N19,H20) |  | 118.27 | 117.98 | 118.28 | 110.72 |  | |
| A(C17,N19,C21) |  | 121.84 | 121.55 | 121.72 | 109.39 |  | |
| A(N19,C21,C24) |  | 110.52 | 110.52 | 111.46 | 109.29 |  | |
| A(O41,P40,O43) |  | 109.70 |  | 109.97 | 109.80 | 108.95 | |
| A(P40,O43,C44) |  | 116.46 |  | 116.85 | 116.79 | 117.62 | |
| D(O11,C2,C3,C4) | 178.63 | -179.89 | -180.00 | 179.93 | -178.54 | -179.58 | |
| D(C17,C3,C4,C5) | 178.82 | 179.74 | 180.00 | -179.84 | 177.39 | 179.39 | |
| D(C3,C4,C5,C6) | -179.21 | -179.88 | 179.96 | -179.98 | -179.13 | -178.81 | |
| D(C4,C5,C6,C7) | -178.47 | 179.84 | 179.87 | -179.99 | 178.24 | 179.84 | |
| D(C4,C5,C10,C9) | 177.82 | -179.85 | -179.87 | 179.99 | -178.09 | 179.96 | |
| D(C6,C5,C10,O1) | 178.15 | 179.99 | -179.95 | -179.98 | -179.24 | 179.59 | |
| D(C7,C8,C9,C10) | 1.83 | -0.01 | -0.06 | -0.06 | 0.04 | 0.00 | |
| D(O18,C17,N19,C21) |  | -0.68 | -0.94 | 0.83 |  |  | |
| D(C36,O39,P40,O41) |  | -175.67 |  | -178.69 | -176.40 |  | |
| D(C36,O39,P40,O42) |  | -39.34 |  | -42.56 | -40.09 |  | |
| D(O39,P40,O43,C44) |  | -167.76 |  | -163.53 | -165.33 |  | |

*Values are mean ± SD triplicate assay*
