# Supplementary material for: DFT and molecular simulation validation of the binding activity of PDEδ inhibitors for repression of oncogenic k-Ras
Source: PLoS One. 2024 Mar 8;19(3):e0300035. doi: 10.1371/journal.pone.0300035 (PMC10923412; doi:10.1371/journal.pone.0300035)
Supplement: S2 Table — (DOCX) [file pone.0300035.s003.docx]

**Table S2:**  Mean absolute errors computed for selected bond lengths (A^o^) and angles (degree) of the selected potential target compounds (**V-IX)** of Coumarin derivative verses 3-((2-Oxo-2*H*-chromen-3-yl)carbonyl)pyridinium hydrogen squarate calculated at long-range corrections wb97xd/6-311++g(d,p) level of theory. The X-ray crystal structure data (CCDC: 697425).

|  | **V** | **VI** | **VII** | **VIII** | **IX** |
| --- | --- | --- | --- | --- | --- |
| R(O1,C2) | 0.064 | 0.068 | 0.063 | 0.053 | 0.055 |
| R(O1,C10) | 0.103 | 0.101 | 0.103 | 0.104 | 0.102 |
| R(C2,C3) | 0.143 | 0.142 | 0.143 | 0.157 | 0.123 |
| R(C2,O11) | 0.124 | 0.122 | 0.125 | 0.135 | 0.126 |
| R(C3,C4) | 0.030 | 0.029 | 0.030 | 0.030 | 0.022 |
| R(C3,C17) | 0.081 | 0.078 | 0.081 | 0.045 | 0.356 |
| R(C4,C5) | 0.038 | 0.037 | 0.038 | 0.043 | 0.057 |
| R(C5,C6) | 0.023 | 0.023 | 0.023 | 0.024 | 0.026 |
| R(C5,C10) | 0.066 | 0.066 | 0.066 | 0.065 | 0.065 |
| R(C6,C7) | 0.068 | 0.067 | 0.068 | 0.069 | 0.069 |
| R(C7,C8) | 0.032 | 0.033 | 0.032 | 0.031 | 0.030 |
| R(C8,C9) | 0.018 | 0.018 | 0.018 | 0.017 | 0.018 |
| R(C9,C10) | 0.097 | 0.096 | 0.097 | 0.097 | 0.098 |
| R(C17,O18) | 0.031 | 0.032 | 0.030 |  |  |
| A(O1,C2,C3) | 1.09 | 1.01 | 1.11 | 1.87 | 0.83 |
| A(C3,C2,O11) | 0.84 | 1.02 | 0.82 | 0.70 | 0.18 |
| A(C4,C3,C17) | 0.54 | 0.67 | 0.55 | 1.38 | 1.69 |
| A(C3,C4,C5) | 1.02 | 0.97 | 1.04 | 1.22 | 1.07 |
| A(C3,C4,H12) | 1.99 | 1.85 | 2.01 | 1.76 | 0.38 |
| A(C5,C4,H12) | 1.03 | 1.11 | 1.03 | 1.59 | 2.32 |
| A(C4,C5,C6) | 1.62 | 1.58 | 1.59 | 1.60 | 1.02 |
| A(C4,C5,C10) | 0.13 | 0.15 | 0.17 | 0.17 | 0.15 |
| A(C7,C6,H13) | 0.13 | 0.05 | 0.04 | 0.33 | 0.13 |
| A(C6,C7,H14) | 0.56 | 0.47 | 0.48 | 0.57 | 0.76 |
| A(C8,C7,H14) | 1.07 | 1.18 | 1.15 | 1.05 | 0.87 |
| A(C7,C8,H15) | 0.84 | 0.80 | 0.87 | 0.99 | 0.96 |
| A(C9,C8,H15) | 0.72 | 0.70 | 0.71 | 0.75 | 0.76 |
| A(C8,C9,C10) | 0.29 | 0.22 | 0.32 | 0.52 | 0.76 |
| A(C10,C9,H16) | 1.54 | 1.41 | 1.50 | 1.65 | 1.92 |
| A(O1,C10,C5) | 0.07 | 0.00 | 0.05 | 0.11 | 1.24 |
| A(C5,C10,C9) | 1.13 | 1.17 | 1.11 | 1.01 | 0.40 |
| D(O11,C2,C3,C4) | 1.26 | 1.37 | 1.29 | 0.09 | 0.95 |
| D(C17,C3,C4,C5) | 0.92 | 1.18 | 1.03 | 1.43 | 0.58 |
| D(C17,C3,C4,H12) | 1.39 | 1.50 | 1.54 | 0.76 | 0.86 |
| D(C3,C4,C5,C6) | 0.67 | 0.75 | 0.76 | 0.09 | 0.40 |
| D(H12,C4,C5,C6) | 1.14 | 1.14 | 0.97 | 0.23 | 0.11 |
| D(C4,C5,C6,C7) | 1.37 | 1.40 | 1.52 | 0.23 | 1.37 |
| D(C4,C5,C6,H13) | 0.92 | 0.83 | 0.97 | 0.59 | 0.87 |
| D(C6,C5,C10,O1) | 1.83 | 1.80 | 1.82 | 1.08 | 1.44 |
| D(C5,C6,C7,C8) | 1.11 | 1.07 | 1.07 | 0.90 | 1.02 |
| D(H13,C6,C7,C8) | 1.55 | 1.56 | 1.57 | 1.25 | 1.36 |
| D(H14,C7,C8,H15) | 1.09 | 1.10 | 1.12 | 1.06 | 1.01 |
| D(C7,C8,C9,C10) | 1.82 | 1.77 | 1.76 | 1.79 | 1.83 |
| D(C8,C9,C10,C5) | 1.07 | 1.07 | 1.03 | 0.76 | 0.82 |
| D(H16,C9,C10,O1) | 1.83 | 1.73 | 1.69 | 1.18 | 1.63 |

*Values are mean ± SD triplicate assay*
