# Supplementary material for: DFT and molecular simulation validation of the binding activity of PDEδ inhibitors for repression of oncogenic k-Ras
Source: PLoS One. 2024 Mar 8;19(3):e0300035. doi: 10.1371/journal.pone.0300035 (PMC10923412; doi:10.1371/journal.pone.0300035)
Supplement: S3 Table — (DOCX) [file pone.0300035.s004.docx]

**Table S3.** The selected bond length (Å), bond angles and dihedral angles, (degree) of selected potential target compounds Deltarazin and Deltaflexin derivatives (**I, X- XI)** using wb97xd/6-311++g(d,p) level of theory.

|  | **I** |  | **I** |  | **X** | **XI** |
| --- | --- | --- | --- | --- | --- | --- |
| R(C1,C2) | 1.399 | A(N60,C61,C73) | 123.43 | R(C1,C2) | 1.415 | 1.415 |
| R(C1,C6) | 1.390 | A(C61,N62,C63) | 105.11 | R(C1,C6) | 1.402 | 1.403 |
| R(C1,C11) | 1.472 | A(N62,C63,C64) | 129.10 | R(C1,C13) | 1.482 | 1.480 |
| R(C4,O38) | 1.356 | A(C64,C63,C68) | 120.54 | R(C2,N10) | 1.372 | 1.369 |
| R(C11,N12) | 1.383 | A(C64,C65,C66) | 121.04 | R(C4,C20) | 1.509 | 1.510 |
| R(C11,N19) | 1.307 | A(N60,C68,C67) | 133.05 | R(C13,O14) | 1.206 | 1.206 |
| R(N12,C24) | 1.451 | A(C63,C68,C67) | 121.63 | R(C13,O15) | 1.354 | 1.356 |
| R(C13,C14) | 1.392 | D(C6,C1,C2,C3) | -1.29 | R(O15,C16) | 1.428 | 1.427 |
| R(C13,C18) | 1.402 | D(C11,C1,C6,C5) | 179.20 | R(C20,O21) | 1.220 | 1.220 |
| R(C18,N19) | 1.382 | D(C6,C1,C11,N12) | 134.14 | R(C20,N22) | 1.352 | 1.351 |
| R(C24,C27) | 1.515 | D(C2,C3,C4,O38) | -179.64 | R(N22,H23) | 1.007 | 1.007 |
| R(C27,C28) | 1.392 | D(C3,C4,O38,C39) | 178.01 | R(N22,C24) | 1.455 | 1.457 |
| R(O38,C39) | 1.413 | D(C1,C11,N12,C13) | -179.05 | R(C33,O36) |  | 1.405 |
| R(C39,C42) | 1.524 | D(C1,C11,N12,C24) | -15.05 | R(O36,P37) | 1.670 | 1.671 |
| R(C42,C44) | 1.537 | D(N19,C11,N12,C13) | 0.86 | R(P37,O38) | 1.489 | 1.489 |
| R(C42,N60) | 1.465 | D(C1,C11,N19,C18) | 179.41 | R(P37,O39) | 1.489 | 1.489 |
| R(C46,N47) | 1.457 | D(N12,C11,N19,C18) | -0.50 | R(P37,O40) | 1.664 | 1.663 |
| R(N60,C61) | 1.385 | D(C11,N12,C13,C14) | 178.00 | R(O40,C41) | 1.407 | 1.408 |
| R(N60,C68) | 1.390 | D(C11,N12,C13,C18) | -0.82 | A(C2,C1,C6) | 119.07 | 119.01 |
| R(C61,N62) | 1.305 | D(C11,N12,C24,C27) | -100.16 | A(C3,C2,N10) | 118.05 | 118.06 |
| R(C61,C73) | 1.475 | D(N12,C13,C14,C15) | -179.83 | A(C3,C4,C20) | 116.90 | 117.00 |
| R(C63,C64) | 1.396 | D(C18,C13,C14,C15) | -1.18 | A(C1,C13,O14) | 124.70 | 124.82 |
| R(C73,C74) | 1.396 | D(N12,C24,C27,C32) | 36.37 | A(C1,C13,O15) | 114.08 | 114.10 |
| A(C1,C2,C3) | 120.83 | D(C30,C31,C32,C27) | -0.01 | A(O14,C13,O15) | 121.21 | 121.09 |
| A(C3,C4,O38) | 115.73 | D(C4,O38,C39,C42) | -176.86 | A(C4,C20,O21) | 121.34 | 121.15 |
| A(C1,C11,N12) | 123.13 | D(O38,C39,C42,N60) | 80.07 | A(O21,C20,N22) | 122.84 | 123.15 |
| A(C1,C11,N19) | 123.64 | D(N60,C42,C44,C45) | 54.36 | A(N22,C24,C27) | 110.53 | 110.55 |
| A(N12,C11,N19) | 113.22 | D(C44,C42,N60,C61) | -107.88 | A(C24,C27,H28) | 109.26 | 109.19 |
| A(C11,N12,C24) | 128.46 | D(C44,C45,C46,N47) | -56.55 | A(C30,C33,C45) | 113.22 | 108.52 |
| A(N12,C13,C18) | 105.53 | D(C42,N60,C61,C73) | -17.82 | A(P37,O36,C48) | 117.66 |  |
| A(C11,N19,C18) | 105.26 | D(C68,N60,C61,C73) | -179.91 | A(C33,O36,P37) |  | 117.53 |
| A(N12,C24,C27) | 113.12 | D(N60,C61,C73,C74) | -47.60 | A(O36,P37,O39) | 108.55 | 108.37 |
| A(C24,C27,C32) | 120.88 | D(N62,C61,C73,C74) | 131.94 | A(P37,O40,C41) | 117.56 | 117.55 |
| A(C30,C31,C32) | 120.23 | D(C61,N62,C63,C64) | 179.76 | A(O36,C48,C45) | 108.59 |  |
| A(C4,O38,C39) | 118.81 | D(C61,N62,C63,C68) | 0.02 | D(C6,C1,C2,C3) | -0.39 | -0.36 |
| A(O38,C39,C42) | 109.93 | D(N62,C63,C64,C65) | 179.66 | D(C6,C1,C2,N10) | 177.56 | 178.11 |
| A(C39,C42,C44) | 114.97 | D(N62,C63,C68,C67) | -179.85 | D(C13,C1,C2,C3) | 178.55 | 178.60 |
| A(C39,C42,N60) | 112.37 | D(C64,C63,C68,C67) | 0.38 | D(C13,C1,C2,N10) | -3.50 | -2.93 |
| A(C44,C42,N60) | 111.81 | D(C63,C64,C65,C66) | 0.40 | D(C2,C1,C13,O15) | -3.60 | -2.45 |
| A(C45,C44,C49) | 110.20 | D(C66,C67,C68,N60) | 179.91 | D(C20,C4,C5,C6) | 179.21 | 179.03 |
| A(C45,C46,N47) | 109.84 | D(C78,C73,C74,C75) | -0.85 | D(C5,C4,C20,N22) | 27.73 | 28.80 |
| A(C44,C49,C48) | 110.86 | D(C73,C74,C75,C76) | 0.18 | D(C20,N22,C24,C27) | 176.27 | 173.80 |
| A(C42,N60,C61) | 124.75 | D(C74,C75,C76,C77) | 0.48 | D(O36,P37,O40,C41) | -71.06 | -70.81 |
| A(N60,C61,N62) | 113.69 | D(C76,C77,C78,C73) | -0.23 | D(O39,P37,O40,C41) | 176.60 | 177.05 |

*Values are mean ± SD triplicate assay*
