# Supplementary material for: DFT and molecular simulation validation of the binding activity of PDEδ inhibitors for repression of oncogenic k-Ras
Source: PLoS One. 2024 Mar 8;19(3):e0300035. doi: 10.1371/journal.pone.0300035 (PMC10923412; doi:10.1371/journal.pone.0300035)
Supplement: S4 Table — (DOCX) [file pone.0300035.s005.docx]

**Table S4.** Natural charge of selected atoms of selected potential eight target compounds (**I,V-XI)** by using wb97xd/6-311++g(d,p) level of theory.

|  | **I** |  | **I** |  | **V** | **VI** | **VII** | **VIII** | **IX** | |  | **X** | **XI** |
| --- | --- | --- | --- | --- | --- | --- | --- | --- | --- | --- | --- | --- | --- |
| **C1** | -0.189 | **C61** | 0.560 | **O1** | -0.508 | -0.505 | -0.508 | -0.520 | -0.523 | | **C1** | -0.232 | -0.236 |
| **C2** | -0.194 | **N62** | -0.531 | **C2** | 0.793 | 0.795 | 0.792 | 0.794 | 0.777 | | **C2** | 0.228 | 0.229 |
| **C3** | -0.190 | **C63** | 0.064 | **C3** | -0.239 | -0.248 | -0.236 | -0.152 | -0.445 | | **C3** | -0.228 | -0.230 |
| **C4** | 0.322 | **C64** | -0.196 | **C4** | -0.053 | -0.043 | -0.056 | -0.108 | 0.431 | | **C4** | -0.074 | -0.070 |
| **C5** | -0.313 | **C65** | -0.224 | **C5** | -0.160 | -0.163 | -0.159 | -0.151 | -0.167 | | **C5** | -0.257 | -0.258 |
| **C6** | -0.080 | **C66** | -0.211 | **C6** | -0.152 | -0.150 | -0.154 | -0.162 | -0.163 | | **C6** | -0.124 | -0.124 |
| **C11** | 0.550 | **C67** | -0.285 | **C7** | -0.225 | -0.222 | -0.226 | -0.227 | -0.205 | | **H7** | 0.233 | 0.232 |
| **N12** | -0.462 | **C68** | 0.256 | **C8** | -0.171 | -0.165 | -0.172 | -0.179 | -0.184 | | **H9** | 0.229 | 0.228 |
| **C13** | 0.135 | **C73** | -0.139 | **C9** | -0.238 | -0.238 | -0.238 | -0.237 | -0.242 | | **N10** | -0.791 | -0.792 |
| **C14** | -0.238 | **C74** | -0.200 | **C10** | 0.358 | 0.358 | 0.358 | 0.354 | 0.353 | | **H11** | 0.410 | 0.412 |
| **C15** | -0.205 | **C75** | -0.203 | **O11** | -0.591 | -0.602 | -0.585 | -0.537 | -0.592 | | **H12** | 0.381 | 0.383 |
| **C16** | -0.224 | **C76** | -0.197 | **H12** | 0.254 | 0.254 | 0.253 | 0.217 | 0.238 | | **C13** | 0.817 | 0.817 |
| **C17** | -0.195 | **C77** | -0.199 | **H13** | 0.218 | 0.220 | 0.217 | 0.210 | 0.232 | | **O14** | -0.614 | -0.616 |
| **C18** | 0.102 | **C78** | -0.166 | **H16** | 0.230 | 0.232 | 0.231 | 0.229 | 0.226 | | **O15** | -0.584 | -0.586 |
| **N19** | -0.534 | **H79** | 0.227 | **C17** | 0.672 | 0.675 | 0.671 | 0.199 |  | | **C16** | -0.212 | -0.212 |
| **C24** | -0.189 |  |  | **O17** |  |  |  |  | -0.535 | | **C20** | 0.675 | 0.674 |
| **H26** | 0.234 |  |  | **O18** | -0.663 | -0.659 | -0.668 |  |  | | **O21** | -0.641 | -0.644 |
| **C27** | -0.048 |  |  | **C18** |  |  |  | -0.646 | -0.025 | | **N22** | -0.627 | -0.624 |
| **C28** | -0.208 |  |  | **N19** | -0.625 | -0.634 | -0.622 | -0.104 |  | | **H23** | 0.392 | 0.392 |
| **C29** | -0.196 |  |  | **H20** | 0.428 | 0.429 | 0.430 | 0.228 | 0.179 | | **C24** | -0.177 | -0.175 |
| **C30** | -0.207 |  |  | **C21** | -0.176 | -0.177 | -0.173 | -0.009 | -0.401 | | **C27** | -0.392 | -0.396 |
| **C31** | -0.190 |  |  | **C24** | -0.393 | -0.392 | -0.385 | -0.405 | -0.384 | | **C30** | -0.377 | -0.398 |
| **C32** | -0.202 |  |  | **C27** | -0.377 | -0.379 | -0.390 | -0.379 | -0.382 | | **C33** | -0.381 | -0.024 |
| **O38** | -0.569 |  |  | **H28** | 0.191 | 0.195 | 0.234 | 0.191 | 0.188 | | **O36** | -0.858 | -0.857 |
| **C39** | -0.030 |  |  | **C30** | -0.381 | -0.382 | -0.381 | -0.380 | -0.397 | | **P37** | 2.458 | 2.458 |
| **C42** | -0.032 |  |  | **C33** | -0.398 | -0.403 | -0.376 | -0.398 | -0.024 | | **O38** | -1.143 | -1.142 |
| **H43** | 0.236 |  |  | **C36** | -0.022 | -0.017 | -0.020 | -0.022 |  | | **O39** | -1.145 | -1.145 |
| **C44** | -0.241 |  |  | **O39** | -0.849 | -0.737 | -0.852 | -0.848 | **O36** | -0.857 | **O40** | -0.856 | -0.856 |
| **C45** | -0.404 |  |  | **P40** | 2.454 | 0.458 | 2.456 | 2.454 | **P37** | 2.458 | **C41** | -0.205 | -0.206 |
| **C46** | -0.173 |  |  | **O41** | -1.136 |  | -1.134 | -1.135 | **O38** | -1.143 | **C45** | -0.397 |  |
| **N47** | -0.685 |  |  | **O42** | -1.163 |  | -1.163 | -1.164 | **O39** | -1.145 | **C48** | -0.023 |  |
| **C48** | -0.173 |  |  | **O43** | -0.857 |  | -0.857 | -0.858 | **O40** | -0.856 |  |  |  |
| **C49** | -0.399 |  |  | **C44** | -0.204 |  | -0.202 | -0.203 | **C41** | -0.205 |  |  |  |
| **H54** | 0.355 |  |  | **H47** | 0.171 |  | 0.172 | 0.157 | **H44** | 0.154 |  |  |  |
| **N60** | -0.502 |  |  | **C48** |  |  | -0.239 |  |  | |  |  |  |
|  |  |  |  | **O48** |  |  |  | -0.438 |  | |  |  |  |

*Values are mean ± SD triplicate assays.*
