# Supplementary material for: DFT and molecular simulation validation of the binding activity of PDEδ inhibitors for repression of oncogenic k-Ras
Source: PLoS One. 2024 Mar 8;19(3):e0300035. doi: 10.1371/journal.pone.0300035 (PMC10923412; doi:10.1371/journal.pone.0300035)
Supplement: S5 Table — (DOCX) [file pone.0300035.s006.docx]

**Table S5.** Values of the Fukui functions and Dual descriptor of selected potential target compounds (**V-XI)** using wb97xd/6-311++g(d,p) level of theory

|  | Deltaflexin-1 **(V)** | | | **VI** | | | **VII** | | | **VIII** | | | **IX** | | | |
| --- | --- | --- | --- | --- | --- | --- | --- | --- | --- | --- | --- | --- | --- | --- | --- | --- |
|  | **f(-)** | **f(+)** | **Δf** | **f(-)** | **f(+)** | **Δf** | **f(-)** | **f(+)** | **Δf** | **f(-)** | **f(+)** | **Δf** | **f(-)** | | **f(+)** | **Δf** |
| **O1** | 0.000 | **0.028** | **0.028** | **0.039** | 0.029 | **-0.010** | 0.000 | **0.028** | **0.028** | 0.000 | **0.025** | **0.025** | 0.000 | | **0.019** | **0.019** |
| **C2** | 0.000 | **0.057** | **0.057** | 0.017 | **0.057** | **0.041** | 0.000 | **0.056** | **0.056** | 0.000 | **0.048** | **0.048** | 0.000 | | **0.057** | **0.057** |
| **C3** | 0.000 | **0.162** | **0.162** | **0.185** | 0.157 | **-0.028** | 0.000 | **0.164** | **0.164** | 0.000 | **0.192** | **0.192** | 0.000 | | **0.146** | **0.146** |
| **C4** | 0.000 | **0.260** | **0.260** | 0.037 | **0.275** | **0.238** | 0.000 | **0.256** | **0.256** | 0.000 | **0.228** | **0.228** | 0.000 | | **0.167** | **0.167** |
| **C5** | 0.000 | **0.040** | **0.040** | **0.160** | 0.035 | **-0.125** | 0.000 | **0.042** | **0.042** | 0.000 | **0.047** | **0.047** | 0.000 | | **0.066** | **0.066** |
| **C6** | 0.000 | **0.099** | **0.099** | 0.008 | **0.095** | **0.087** | 0.000 | **0.099** | **0.099** | 0.000 | **0.095** | **0.095** | 0.000 | | **0.151** | **0.151** |
| **C8** | 0.000 | **0.132** | **0.132** | 0.121 | **0.127** | **0.006** | 0.000 | **0.133** | **0.133** | 0.000 | **0.128** | **0.128** | 0.000 | | **0.164** | **0.164** |
| **C9** | 0.000 | **0.042** | **0.042** | 0.004 | **0.036** | **0.033** | 0.000 | **0.044** | **0.044** | 0.000 | **0.044** | **0.044** | 0.000 | | **0.085** | **0.085** |
| **C10** | 0.000 | **0.061** | **0.061** | **0.132** | 0.065 | **-0.067** | 0.000 | **0.061** | **0.061** | 0.000 | **0.052** | **0.052** | 0.000 | | **0.047** | **0.047** |
| **O11** | 0.000 | **0.040** | **0.040** | **0.122** | 0.039 | **-0.084** | 0.000 | **0.040** | **0.040** | 0.000 | **0.043** | **0.043** | 0.000 | | **0.041** | **0.041** |
| **C17** | 0.000 | **0.025** | **0.025** | 0.003 | **0.029** | **0.026** | 0.000 | **0.024** | **0.024** | 0.000 | **0.008** | **0.008** |  | |  |  |
| **O17** |  |  |  |  |  |  |  |  |  |  |  |  | 0.000 | | **0.033** | **0.033** |
| **O18** | 0.000 | **0.019** | **0.019** | 0.011 | **0.022** | **0.011** | 0.000 | **0.019** | **0.019** |  |  |  |  | |  |  |
| **N19** | 0.000 | 0.020 | 0.020 | 0.037 | 0.020 | -0.017 | 0.000 | 0.021 | 0.021 | 0.000 | 0.054 | 0.054 |  | |  |  |
| **C27** | 0.000 | 0.000 | 0.000 | 0.000 | 0.001 | 0.001 | -0.002 | 0.000 | 0.002 | 0.000 | 0.001 | 0.001 | 0.007 | | -0.001 | -0.008 |
| **C30** | 0.010 | 0.000 | -0.010 | 0.000 | 0.000 | 0.000 | 0.002 | 0.000 | -0.002 | 0.008 | 0.000 | -0.008 | 0.013 | | 0.000 | -0.013 |
| **C33** | 0.006 | 0.000 | -0.006 | 0.000 | 0.000 | 0.000 | 0.001 | 0.000 | -0.001 | 0.006 | 0.000 | -0.006 | -0.015 | | 0.000 | 0.014 |
| **C36** | -0.006 | 0.000 | 0.006 | 0.000 | 0.000 | 0.000 | -0.012 | 0.000 | 0.012 | -0.005 | 0.000 | 0.005 |  | |  |  |
| **O36** |  |  |  |  |  |  |  |  |  |  |  |  | **0.023** | | 0.000 | **-0.023** |
| **O39** | **0.007** | 0.000 | **-0.007** | 0.000 | 0.000 | 0.000 | **0.006** | 0.000 | **-0.006** | **0.008** | 0.000 | **-0.008** |  | |  |  |
| **P40** | **0.045** | 0.000 | **-0.045** | 0.000 | 0.000 | 0.000 | **0.056** | 0.000 | **-0.056** | **0.045** | 0.000 | **-0.045** | **P37** | **0.058** | 0.000 | **-0.058** |
| **O41** | **0.414** | 0.000 | **-0.414** |  |  |  | **0.418** | 0.000 | **-0.418** | **0.419** | 0.000 | **-0.419** | **O38** | **0.435** | 0.000 | **-0.435** |
| **O42** | **0.380** | 0.000 | **-0.380** |  |  |  | **0.372** | 0.000 | **-0.372** | **0.377** | 0.000 | **-0.377** | **O39** | **0.431** | 0.000 | **-0.431** |
| **O43** | **0.116** | 0.000 | **-0.116** |  |  |  | **0.113** | 0.000 | **-0.113** | **0.113** | 0.000 | **-0.113** | **O40** | **0.027** | 0.000 | **-0.027** |
| **C44** | **0.002** | 0.000 | **-0.002** |  |  |  | **0.004** | 0.000 | **-0.004** | **0.002** | 0.000 | **-0.002** | **C41** | **0.004** | 0.000 | **-0.004** |
| **H47** | **0.012** | 0.000 | **-0.012** |  |  |  | **0.011** | 0.000 | **-0.011** | **0.008** | 0.000 | **-0.008** | **H44** | **-0.003** | 0.000 | **0.003** |
| **O48** |  |  |  |  |  |  |  |  |  | 0.000 | **0.012** | **0.012** |  | |  |  |

*Values are mean ± SD triplicate assays.*
