# Supplementary material for: DFT and molecular simulation validation of the binding activity of PDEδ inhibitors for repression of oncogenic k-Ras
Source: PLoS One. 2024 Mar 8;19(3):e0300035. doi: 10.1371/journal.pone.0300035 (PMC10923412; doi:10.1371/journal.pone.0300035)
Supplement: S6 Table — (DOCX) [file pone.0300035.s007.docx]

**Table S6.** Values of the Fukui functions and Dual descriptor of selected potential target compounds (**I, X-XI**) using wb97xd/6-311++g(d,p) level of theory.

|  | **Deltarazin (I)** | | | | | | |  | **Deltaflexin-2 (X)** | | | **XI** | | |
| --- | --- | --- | --- | --- | --- | --- | --- | --- | --- | --- | --- | --- | --- | --- |
| **Atoms** | **f(-)** | **f(+)** | **Δf** | **Atoms** | **f(-)** | **f(+)** | **Δf** | **Atoms** | **f(-)** | **f(+)** | **Δf** | **f(-)** | **f(+)** | **Δf** |
| **C1** | **0.059** | 0.000 | **-0.059** | **C42** | -0.003 | 0.027 | 0.030 | **C1** | 0.000 | **0.136** | **0.136** | 0.000 | **0.125** | **0.125** |
| **C2** | **0.028** | 0.000 | **-0.028** | **H43** | 0.000 | -0.003 | -0.003 | **C2** | 0.000 | **0.042** | **0.042** | 0.000 | **0.047** | **0.047** |
| **C3** | **0.028** | 0.001 | **-0.028** | **C44** | 0.000 | -0.026 | -0.026 | **C3** | 0.000 | **0.083** | **0.083** | 0.000 | **0.084** | **0.084** |
| **C4** | **0.062** | -0.001 | **-0.063** | **C45** | 0.000 | -0.001 | -0.001 | **C4** | 0.000 | **0.176** | **0.176** | 0.000 | **0.176** | **0.176** |
| **C5** | **0.026** | -0.003 | **-0.028** | **C46** | 0.000 | -0.004 | -0.004 | **C5** | 0.000 | **0.023** | **0.023** | 0.000 | **0.021** | **0.021** |
| **C6** | **0.040** | 0.002 | **-0.038** | **N47** | 0.000 | 0.001 | 0.001 | **C6** | 0.000 | **0.132** | **0.132** | 0.000 | **0.137** | **0.137** |
| **C11** | **0.117** | 0.000 | **-0.117** | **C48** | 0.000 | -0.003 | -0.003 | **N10** | 0.000 | **0.022** | **0.022** | 0.000 | **0.023** | **0.023** |
| **N12** | 0.000 | 0.000 | 0.000 | **C49** | 0.001 | -0.004 | -0.005 | **C13** | 0.000 | **0.150** | **0.150** | 0.000 | **0.158** | **0.158** |
| **C13** | **0.117** | 0.000 | **-0.117** | **N60** | 0.000 | **0.013** | **0.013** | **O14** | 0.000 | **0.080** | **0.080** | 0.000 | **0.083** | **0.083** |
| **C14** | **0.015** | 0.000 | **-0.015** | **C61** | 0.000 | **0.105** | **0.105** | **O15** | 0.000 | **0.021** | **0.021** | 0.000 | **0.021** | **0.021** |
| **C15** | **0.158** | 0.000 | **-0.158** | **N62** | 0.000 | **0.060** | **0.060** | **C16** | 0.000 | **0.003** | **0.003** | 0.000 | **0.003** | **0.003** |
| **C16** | **0.044** | 0.000 | **-0.044** | **C63** | 0.001 | 0.005 | 0.004 | **C20** | 0.000 | **0.053** | **0.053** | 0.000 | **0.050** | **0.050** |
| **C17** | **0.049** | 0.000 | **-0.049** | **C64** | 0.000 | **0.076** | **0.075** | **O21** | 0.000 | **0.039** | **0.039** | 0.000 | **0.036** | **0.036** |
| **C18** | **0.118** | 0.000 | **-0.118** | **C65** | 0.001 | 0.011 | 0.010 | **N22** | 0.000 | **0.024** | **0.024** | 0.000 | **0.023** | **0.023** |
| **N19** | **0.077** | 0.000 | **-0.077** | **C66** | 0.001 | **0.049** | **0.048** | **C24** | 0.000 | 0.002 | 0.002 | 0.000 | 0.002 | 0.002 |
| **C24** | -0.002 | -0.001 | 0.001 | **C67** | 0.001 | **0.058** | **0.058** | **C30** | 0.001 | 0.000 | 0.000 | 0.010 | 0.000 | -0.010 |
| **C27** | 0.004 | 0.000 | -0.004 | **C68** | 0.001 | **0.036** | **0.036** | **C33** | 0.010 | 0.000 | -0.010 | -0.009 | 0.000 | 0.009 |
| **C28** | -0.003 | 0.001 | 0.003 | **C73** | 0.001 | **0.154** | **0.153** | **O36** | **0.024** | 0.000 | **-0.024** | **0.021** | 0.000 | **-0.021** |
| **C29** | 0.001 | 0.000 | -0.001 | **C74** | 0.000 | **0.101** | **0.101** | **P37** | **0.057** | 0.000 | **-0.057** | **0.060** | 0.000 | **-0.060** |
| **C30** | 0.001 | 0.000 | -0.002 | **C75** | 0.000 | **0.036** | **0.036** | **O38** | **0.434** | 0.000 | **-0.434** | **0.438** | 0.000 | **-0.438** |
| **C31** | -0.003 | 0.000 | 0.003 | **C76** | 0.000 | **0.171** | **0.171** | **O39** | **0.431** | 0.000 | **-0.431** | **0.429** | 0.000 | **-0.429** |
| **C32** | 0.002 | 0.000 | -0.003 | **C77** | 0.000 | **0.052** | **0.052** | **O40** | **0.028** | 0.000 | **-0.028** | **0.029** | 0.000 | **-0.029** |
| **O38** | 0.042 | 0.001 | -0.041 | **C78** | 0.000 | **0.066** | **0.065** | **C41** | **0.003** | 0.000 | **-0.003** | **0.004** | 0.000 | **-0.004** |
| **C39** | 0.000 | -0.011 | -0.011 |  |  |  |  | **C45** | **0.016** | 0.000 | **-0.016** |  |  |  |
|  |  |  |  |  |  |  |  | **C48** | **-0.019** | 0.000 | **0.019** |  |  |  |

*Values are mean ± SD triplicate assay.*
