# Supplementary material for: DFT and molecular simulation validation of the binding activity of PDEδ inhibitors for repression of oncogenic k-Ras
Source: PLoS One. 2024 Mar 8;19(3):e0300035. doi: 10.1371/journal.pone.0300035 (PMC10923412; doi:10.1371/journal.pone.0300035)
Supplement: S7 Table — (DOCX) [file pone.0300035.s008.docx]

**Table S7:** Values of the Condensed local Softnesses (Hartree*e) of selected potential target compounds (**V-IX**) by using wb97xd/6-311++g(d,p) level of theory from CDFT point of view.

|  | Deltaflexin-1 **(V)** | | **VI** | | **VII** | | **VIII** | | **IX** | |
| --- | --- | --- | --- | --- | --- | --- | --- | --- | --- | --- |
|  | **s+/s-** | **s-/s+** | **s+/s-** | **s-/s+** | **s+/s-** | **s-/s+** | **s+/s-** | **s-/s+** | **s+/s-** | **s-/s+** |
| **O1** | 0.037 | 27.273 | 0.953 | 1.049 | 0.033 | 30.183 | 0.044 | 22.855 | 0.111 | 9.036 |
| **C2** | -0.012 | -86.412 | 0.620 | 1.613 | -0.017 | -59.311 | -0.006 | -159.275 | 0.053 | 18.980 |
| **C3** | -0.043 | -23.230 | 1.057 | 0.946 | -0.051 | -19.508 | -0.034 | -29.357 | 0.038 | 26.540 |
| **C4** | 0.037 | 26.797 | 0.315 | 3.172 | 0.050 | 20.013 | 0.036 | 28.090 | -0.035 | -28.636 |
| **C5** | -0.022 | -45.844 | 3.798 | 0.263 | -0.014 | -70.456 | -0.037 | -26.729 | -0.049 | -20.538 |
| **C6** | 0.041 | 24.698 | 0.627 | 1.594 | 0.056 | 17.783 | 0.030 | 33.786 | -0.076 | -13.215 |
| **C7** | 0.102 | 9.831 | 1.822 | 0.549 | 0.127 | 7.849 | 0.086 | 11.592 | -0.026 | -38.122 |
| **C8** | 0.062 | 16.199 | 1.078 | 0.928 | 0.072 | 13.894 | 0.059 | 16.922 | 0.038 | 26.090 |
| **C9** | 0.035 | 28.452 | 0.760 | 1.316 | 0.033 | 30.394 | 0.043 | 23.516 | 0.085 | 11.770 |
| **C10** | 0.016 | 63.824 | 1.944 | 0.515 | 0.010 | 96.973 | 0.025 | 39.479 | 0.081 | 12.354 |
| **O11** | -0.078 | -12.771 | 1.396 | 0.717 | -0.123 | -8.119 | -0.037 | -26.726 | 0.105 | 9.536 |
| **H12** | 0.038 | 26.229 | 0.570 | 1.755 | 0.059 | 16.958 | 0.016 | 63.354 | 0.028 | 36.064 |
| **H16** | 0.044 | 22.530 | 0.987 | 1.014 | 0.039 | 25.543 | 0.056 | 17.717 | 0.120 | 8.311 |
| **C17** | 0.086 | 11.609 | 0.292 | 3.421 | 0.103 | 9.753 | 0.569 | 1.757 | -0.160 | -6.239 |
| **O18** | 0.080 | 12.463 | 0.808 | 1.238 | 0.142 | 7.066 |  |  |  |  |
| **C18** |  |  |  |  |  |  | 0.082 | 12.227 | 0.225 | 4.451 |
| **N19** | -0.134 | -7.483 | 1.477 | 0.677 | -0.196 | -5.107 | -0.077 | -13.075 |  |  |
| **H19** |  |  |  |  |  |  |  |  | 0.141 | 7.081 |
| **H20** | -0.078 | -12.875 | 1.040 | 0.961 | -0.199 | -5.026 | -0.002 | -464.858 | 0.448 | 2.231 |
| **C21** | 0.153 | 6.521 | 1.226 | 0.816 | 0.249 | 4.017 | 0.213 | 4.702 | 0.582 | 1.718 |
| **C24** | 1.641 | 0.609 | 0.875 | 1.144 | -2.932 | -0.341 | 0.947 | 1.056 | -8.084 | -0.124 |
| **C27** | 1.169 | 0.856 | 1.018 | 0.982 | -0.015 | -66.980 | 1.440 | 0.695 | 1.426 | 0.701 |
| **C30** | 1.987 | 0.503 | 1.034 | 0.967 | 0.902 | 1.109 | 1.842 | 0.543 | 8.788 | 0.114 |
| **C33** | 3.882 | 0.258 | 1.040 | 0.962 | 1.638 | 0.610 | 4.894 | 0.204 | 7.470 | 0.134 |
| **C36** | 9.723 | 0.103 | 0.992 | 1.008 | 5.451 | 0.184 | 10.125 | 0.099 |  |  |
| **O39** | 31.609 | 0.032 | 1.034 | 0.967 | -17.244 | -0.058 | 51.760 | 0.019 | 341.651 | 0.003 |
| **P40** | 76.249 | 0.013 | 0.991 | 1.009 | 108.775 | 0.009 | 90.574 | 0.011 | 42.478 | 0.024 |
| **O41** | 52.553 | 0.019 |  |  | 75.114 | 0.013 | 61.506 | 0.016 | 48.477 | 0.021 |
| **O42** | 188.552 | 0.005 |  |  | 62.653 | 0.016 | 187.289 | 0.005 | 63.782 | 0.016 |
| **O43** | -66.172 | -0.015 |  |  | -44.835 | -0.022 | -75.648 | -0.013 | 44.117 | 0.023 |
| **C44** | 25.762 | 0.039 |  |  | 17.886 | 0.056 | 26.859 | 0.037 | 60.992 | 0.016 |
| **C48** |  |  |  |  | 1.329 | 0.753 | -0.123 | -8.108 |  |  |
| **C50** |  |  |  |  | -4.081 | -0.245 | 0.403 | 2.479 |  |  |

*Values are mean ± SD triplicate assays*
