# Supplementary material for: DFT and molecular simulation validation of the binding activity of PDEδ inhibitors for repression of oncogenic k-Ras
Source: PLoS One. 2024 Mar 8;19(3):e0300035. doi: 10.1371/journal.pone.0300035 (PMC10923412; doi:10.1371/journal.pone.0300035)
Supplement: S8 Table — (DOCX) [file pone.0300035.s009.docx]

**Table S8:** Values of the Condensed local Softnesses (Hartree*e) of selected potential target compounds (**I, X, XI**) by using wb97xd/6-311++g(d,p) level of theory from CDFT point of view.

|  | **Deltarazin (I)** | | | | |  | **Deltaflexin-2 (X)** | | **XI** | |
| --- | --- | --- | --- | --- | --- | --- | --- | --- | --- | --- |
|  | **s+/s-** | **s-/s+** |  | **s+/s-** | **s-/s+** |  | **s+/s-** | **s-/s+** | **s+/s-** | **s-/s+** |
| **C1** | 1.883 | 0.531 | **N47** | -0.351 | -2.846 | **C1** | 0.056 | 17.887 | 0.085 | 11.753 |
| **C2** | 2.616 | 0.382 | **C48** | 0.169 | 5.904 | **C2** | 0.048 | 20.809 | 0.069 | 14.415 |
| **C3** | -73.771 | -0.014 | **C49** | -0.661 | -1.514 | **C3** | 0.022 | 45.985 | 0.034 | 29.082 |
| **C4** | -4.286 | -0.233 | **N60** | -0.339 | -2.949 | **C4** | -0.041 | -24.588 | -0.058 | -17.175 |
| **C5** | -42.756 | -0.023 | **C61** | 0.080 | 12.517 | **C5** | -0.086 | -11.675 | -0.120 | -8.341 |
| **C6** | 4.295 | 0.233 | **N62** | 0.093 | 10.757 | **C6** | 0.014 | 73.499 | 0.025 | 40.489 |
| **H7** | 6.329 | 0.158 | **C63** | 0.502 | 1.993 | **H7** | 0.023 | 42.992 | 0.042 | 24.116 |
| **C11** | -8.850 | -0.113 | **C64** | 0.142 | 7.050 | **H9** | 0.029 | 33.977 | 0.047 | 21.484 |
| **N12** | 2.664 | 0.375 | **C65** | 0.094 | 10.605 | **N10** | 0.085 | 11.765 | 0.122 | 8.203 |
| **C13** | -119.401 | -0.008 | **C66** | -0.041 | -24.619 | **H11** | 0.111 | 9.029 | 0.154 | 6.497 |
| **C14** | 7.930 | 0.126 | **C67** | -0.290 | -3.455 | **H12** | 0.064 | 15.676 | 0.091 | 11.010 |
| **C15** | 9.754 | 0.103 | **C68** | -0.343 | -2.915 | **C13** | 0.013 | 77.538 | 0.019 | 52.449 |
| **C16** | 5.523 | 0.181 | **C73** | -0.067 | -15.027 | **O14** | 0.029 | 34.305 | 0.045 | 22.439 |
| **C17** | 8.951 | 0.112 | **C74** | -0.107 | -9.389 | **O15** | 0.035 | 28.589 | 0.047 | 21.307 |
| **C18** | -97.756 | -0.010 | **C75** | -0.033 | -30.008 | **C16** | 0.076 | 13.242 | 0.103 | 9.721 |
| **N19** | 10.849 | 0.092 | **C76** | 0.043 | 23.380 | **C20** | 0.062 | 16.220 | 0.113 | 8.885 |
| **H20** | 284.239 | 0.004 | **C77** | 0.080 | 12.510 | **O21** | 0.074 | 13.528 | 0.133 | 7.547 |
| **H23** | -60.386 | -0.017 | **C78** | 0.027 | 36.947 | **N22** | -0.106 | -9.452 | -0.158 | -6.320 |
| **C24** | -2.657 | -0.376 | **H79** | 0.168 | 5.943 | **H23** | -0.018 | -56.863 | -0.002 | -424.403 |
| **C27** | 33.437 | 0.030 |  |  |  | **C24** | 0.171 | 5.861 | 0.359 | 2.788 |
| **C28** | 1.331 | 0.751 |  |  |  | **C27** | 1.319 | 0.758 | 2.173 | 0.460 |
| **C29** | 2.401 | 0.417 |  |  |  | **C30** | 1.231 | 0.812 | 2.284 | 0.438 |
| **C30** | 3.069 | 0.326 |  |  |  | **C33** | 2.285 | 0.438 | 7.029 | 0.142 |
| **C31** | 2.362 | 0.423 |  |  |  | **O36** | 39.867 | 0.025 | 21.073 | 0.048 |
| **C32** | 8.493 | 0.118 |  |  |  | **P37** | 61.298 | 0.016 | 40.585 | 0.025 |
| **O38** | -9.177 | -0.109 |  |  |  | **O38** | 55.236 | 0.018 | 38.075 | 0.026 |
| **C39** | -24.290 | -0.041 |  |  |  | **O39** | 187.347 | 0.005 | 128.865 | 0.008 |
| **C42** | 11.045 | 0.091 |  |  |  | **O40** | 246.957 | 0.004 | 165.159 | 0.006 |
| **C44** | -1.611 | -0.621 |  |  |  | **C41** | 37.751 | 0.027 | 23.935 | 0.042 |
| **C45** | -0.546 | -1.833 |  |  |  | **C45** | 4.196 | 0.238 |  |  |
| **C46** | 0.576 | 1.736 |  |  |  | **C48** | 12.351 | 0.081 |  |  |

*Values are mean ± SD triplicate assays*
