# Supplementary material for: DFT and molecular simulation validation of the binding activity of PDEδ inhibitors for repression of oncogenic k-Ras
Source: PLoS One. 2024 Mar 8;19(3):e0300035. doi: 10.1371/journal.pone.0300035 (PMC10923412; doi:10.1371/journal.pone.0300035)
Supplement: S9 Table — (DOCX) [file pone.0300035.s010.docx]

**Table S9:** Values of the Condensed local electrophilicity (ElP)/nucleophilicity (NuP) index (e*eV) of selected potential target compounds (**V-IX**) by using wb97xd/6-311++g(d,p) level of theory from CDFT point of view.

|  | Deltaflexin-1 **(V)** | | **VI** | | **VII** | | **VIII** | | **IX** | |
| --- | --- | --- | --- | --- | --- | --- | --- | --- | --- | --- |
|  | ElP | NuP | ElP | NuP | ElP | NuP | ElP | NuP | ElP | NuP |
| **O1** | -0.001 | -0.181 | -0.057 | -0.007 | -0.001 | -0.176 | -0.001 | -0.167 | -0.001 | -0.143 |
| **C2** | 0.000 | -0.239 | -0.047 | -0.008 | 0.000 | -0.232 | 0.000 | -0.216 | -0.001 | -0.234 |
| **C3** | 0.002 | -0.400 | -0.134 | -0.014 | 0.002 | -0.396 | 0.001 | -0.404 | -0.001 | -0.344 |
| **C4** | -0.002 | -0.517 | -0.054 | -0.019 | -0.003 | -0.502 | -0.002 | -0.471 | 0.001 | -0.341 |
| **C5** | 0.000 | -0.095 | -0.104 | -0.003 | 0.000 | -0.095 | 0.000 | -0.097 | 0.001 | -0.155 |
| **C6** | -0.001 | -0.258 | -0.050 | -0.009 | -0.001 | -0.254 | -0.001 | -0.251 | 0.002 | -0.312 |
| **C7** | -0.002 | -0.184 | -0.107 | -0.006 | -0.002 | -0.180 | -0.001 | -0.178 | 0.000 | -0.181 |
| **C8** | -0.003 | -0.376 | -0.128 | -0.013 | -0.003 | -0.369 | -0.002 | -0.365 | -0.001 | -0.393 |
| **C9** | -0.001 | -0.205 | -0.047 | -0.007 | -0.001 | -0.202 | -0.001 | -0.201 | -0.002 | -0.257 |
| **C10** | 0.000 | -0.144 | -0.093 | -0.005 | 0.000 | -0.141 | 0.000 | -0.134 | -0.001 | -0.123 |
| **O11** | 0.003 | -0.382 | -0.169 | -0.013 | 0.005 | -0.374 | 0.001 | -0.390 | -0.003 | -0.393 |
| **H12** | -0.001 | -0.210 | -0.039 | -0.008 | -0.001 | -0.204 | 0.000 | -0.205 | 0.000 | -0.181 |
| **H16** | -0.001 | -0.141 | -0.043 | -0.005 | -0.001 | -0.138 | -0.001 | -0.137 | -0.001 | -0.157 |
| **C17** | -0.001 | -0.107 | -0.011 | -0.004 | -0.001 | -0.102 | -0.002 | -0.037 | 0.001 | -0.130 |
| **O18** | -0.002 | -0.216 | -0.058 | -0.008 | -0.003 | -0.208 |  |  |  |  |
| **C18** |  |  |  |  |  |  | 0.000 | -0.047 | -0.001 | -0.037 |
| **N19** | 0.002 | -0.111 | -0.052 | -0.004 | 0.002 | -0.111 | 0.002 | -0.217 |  |  |
| **H19** |  |  |  |  |  |  |  |  | -0.001 | -0.053 |
| **H20** | 0.000 | -0.051 | -0.018 | -0.002 | 0.001 | -0.045 | 0.000 | -0.080 | -0.002 | -0.051 |
| **C21** | -0.001 | -0.037 | -0.015 | -0.001 | -0.001 | -0.033 | -0.001 | -0.030 | -0.001 | -0.017 |
| **C24** | -0.001 | -0.006 | -0.002 | 0.000 | -0.002 | 0.006 | -0.001 | -0.010 | -0.001 | 0.002 |
| **C27** | -0.001 | -0.011 | -0.004 | 0.000 | 0.000 | -0.008 | -0.001 | -0.009 | -0.001 | -0.010 |
| **C30** | -0.001 | -0.007 | -0.002 | 0.000 | -0.001 | -0.013 | -0.001 | -0.007 | -0.002 | -0.003 |
| **C33** | -0.003 | -0.007 | -0.003 | 0.000 | -0.002 | -0.011 | -0.003 | -0.005 | -0.003 | -0.006 |
| **C36** | -0.004 | -0.004 | -0.003 | 0.000 | -0.004 | -0.006 | -0.004 | -0.004 |  |  |
| **O39** | -0.014 | -0.004 | -0.006 | -0.001 | -0.013 | 0.007 | -0.012 | -0.003 | -0.012 | -0.001 |
| **P40** | -0.063 | -0.008 | -0.007 | -0.001 | -0.057 | -0.005 | -0.054 | -0.006 | -0.040 | -0.014 |
| **O41** | -0.139 | -0.024 |  |  | -0.128 | -0.017 | -0.119 | -0.021 | -0.082 | -0.024 |
| **O42** | -0.129 | -0.006 |  |  | -0.115 | -0.018 | -0.109 | -0.006 | -0.083 | -0.019 |
| **O43** | -0.048 | 0.007 |  |  | -0.043 | 0.009 | -0.040 | 0.006 | -0.013 | -0.004 |
| **C44** | -0.013 | -0.005 |  |  | -0.012 | -0.007 | -0.011 | -0.004 | -0.007 | -0.002 |
| **C48** |  |  |  |  | -0.001 | -0.005 | 0.002 | -0.140 |  |  |
| **C50** |  |  |  |  | -0.001 | 0.002 | -0.001 | -0.039 |  |  |

*Values are mean ± SD triplicate assays*
