# Supplementary material for: DFT and molecular simulation validation of the binding activity of PDEδ inhibitors for repression of oncogenic k-Ras
Source: PLoS One. 2024 Mar 8;19(3):e0300035. doi: 10.1371/journal.pone.0300035 (PMC10923412; doi:10.1371/journal.pone.0300035)
Supplement: S10 Table — (DOCX) [file pone.0300035.s011.docx]

**Table S10:** Values of the Condensed local electrophilicity (ElP)/nucleophilicity (NuP) index (e*eV) of selected potential target compounds (**I, X-XI**) by using wb97xd/6-311++g(d,p) level of theory from CDFT point of view.

|  | **Deltarazin (I)** | | | | |  | **Deltaflexin-2 (X)** | | **XI** | |
| --- | --- | --- | --- | --- | --- | --- | --- | --- | --- | --- |
|  | ElP | NuP |  | ElP | NuP |  | ElP | NuP | ElP | NuP |
| **C1** | -0.005 | -0.013 | **N47** | 0.001 | -0.011 | **C1** | -0.001 | -0.233 | -0.001 | -0.223 |
| **C2** | -0.005 | -0.010 | **C48** | 0.000 | -0.006 | **C2** | -0.001 | -0.190 | -0.001 | -0.187 |
| **C3** | -0.007 | 0.000 | **C49** | 0.001 | -0.005 | **C3** | 0.000 | -0.257 | -0.001 | -0.251 |
| **C4** | -0.010 | 0.013 | **N60** | 0.002 | -0.030 | **C4** | 0.001 | -0.355 | 0.001 | -0.351 |
| **C5** | -0.006 | 0.001 | **C61** | -0.001 | -0.053 | **C5** | 0.001 | -0.155 | 0.001 | -0.150 |
| **C6** | -0.006 | -0.008 | **N62** | -0.001 | -0.067 | **C6** | 0.000 | -0.278 | 0.000 | -0.279 |
| **H7** | -0.004 | -0.003 | **C63** | -0.001 | -0.010 | **H7** | 0.000 | -0.161 | 0.000 | -0.160 |
| **C11** | -0.012 | 0.007 | **C64** | -0.001 | -0.054 | **H9** | 0.000 | -0.148 | 0.000 | -0.146 |
| **N12** | -0.002 | -0.005 | **C65** | -0.001 | -0.041 | **N10** | -0.001 | -0.147 | -0.001 | -0.144 |
| **C13** | -0.012 | 0.001 | **C66** | 0.000 | -0.061 | **H11** | -0.001 | -0.097 | -0.001 | -0.096 |
| **C14** | -0.006 | -0.004 | **C67** | 0.002 | -0.044 | **H12** | -0.001 | -0.121 | -0.001 | -0.120 |
| **C15** | -0.019 | -0.011 | **C68** | 0.001 | -0.010 | **C13** | 0.000 | -0.338 | 0.000 | -0.344 |
| **C16** | -0.010 | -0.010 | **C73** | 0.001 | -0.074 | **O14** | -0.001 | -0.395 | -0.001 | -0.397 |
| **C17** | -0.010 | -0.006 | **C74** | 0.001 | -0.071 | **O15** | 0.000 | -0.119 | 0.000 | -0.120 |
| **C18** | -0.013 | 0.001 | **C75** | 0.000 | -0.054 | **C16** | 0.000 | -0.078 | -0.001 | -0.078 |
| **N19** | -0.013 | -0.007 | **C76** | -0.001 | -0.115 | **C20** | -0.001 | -0.148 | -0.001 | -0.134 |
| **H20** | -0.007 | 0.000 | **C77** | -0.001 | -0.063 | **O21** | -0.001 | -0.250 | -0.002 | -0.237 |
| **H23** | -0.006 | 0.001 | **C78** | 0.000 | -0.062 | **N22** | 0.001 | -0.100 | 0.001 | -0.097 |
| **C24** | -0.001 | 0.003 | **H79** | -0.001 | -0.034 | **H23** | 0.000 | -0.050 | 0.000 | -0.047 |
| **C27** | 0.003 | 0.000 |  |  |  | **C24** | 0.000 | -0.034 | -0.001 | -0.033 |
| **C28** | -0.001 | -0.003 |  |  |  | **C27** | -0.001 | -0.007 | -0.001 | -0.007 |
| **C29** | -0.003 | -0.006 |  |  |  | **C30** | -0.001 | -0.010 | -0.002 | -0.012 |
| **C30** | -0.003 | -0.006 |  |  |  | **C33** | -0.001 | -0.006 | -0.003 | -0.006 |
| **C31** | -0.001 | -0.003 |  |  |  | **O36** | -0.013 | -0.004 | -0.011 | -0.008 |
| **C32** | 0.003 | 0.002 |  |  |  | **P37** | -0.044 | -0.010 | -0.038 | -0.014 |
| **O38** | -0.007 | 0.004 |  |  |  | **O38** | -0.091 | -0.022 | -0.080 | -0.031 |
| **C39** | -0.001 | 0.000 |  |  |  | **O39** | -0.092 | -0.007 | -0.080 | -0.009 |
| **C42** | 0.000 | 0.000 |  |  |  | **O40** | -0.015 | -0.001 | -0.013 | -0.001 |
| **C44** | 0.001 | -0.002 |  |  |  | **C41** | -0.008 | -0.003 | -0.007 | -0.004 |
| **C45** | 0.000 | 0.002 |  |  |  | **C45** | -0.002 | -0.006 |  |  |
| **C46** | 0.000 | -0.004 |  |  |  | **C48** | -0.003 | -0.003 |  |  |

*Values are mean ± SD triplicate assays*
